# GMAP is an Atg8a-interacting protein that regulates Golgi turnover in *Drosophila*

**DOI:** 10.1016/j.celrep.2022.110903

**Published:** 2022-05-31

**Authors:** Ashrafur Rahman, Peter Lőrincz, Raksha Gohel, Anikó Nagy, Gábor Csordás, Yan Zhang, Gábor Juhász, Ioannis P. Nezis

**Affiliations:** 1 School of Life Sciences, University of Warwick, CV4 7AL Coventry, UK; 2 Department of Anatomy, Cell and Developmental Biology, Eötvös Loránd University, Budapest, Hungary; 3 Institute of Genetics, Biological Research Centre, Szeged, Hungary; 4 State Key Laboratory of Silkworm Genome Biology, Biological Science Research Center, Southwest University, Chongqing 400715, China

**Keywords:** autophagy, LIR motif, LIR motif docking site, Golgi, Golgiphagy *Drosophila*

## Abstract

Selective autophagy receptors and adapters contain short linear motifs called LIR motifs (LC3-interacting region), which are required for the interaction with the Atg8-family proteins. LIR motifs bind to the hydrophobic pockets of the LIR motif docking site (LDS) of the respective Atg8-family proteins. The physiological significance of LDS docking sites has not been clarified *in vivo*. Here, we show that Atg8a-LDS mutant *Drosophila* flies accumulate autophagy substrates and have reduced lifespan. Using quantitative proteomics to identify the proteins that accumulate in Atg8a-LDS mutants, we identify the *cis*-Golgi protein GMAP (Golgi microtubule-associated protein) as a LIR motif-containing protein that interacts with Atg8a. GMAP LIR mutant flies exhibit accumulation of Golgi markers and elongated Golgi morphology. Our data suggest that GMAP mediates the turnover of Golgi by selective autophagy to regulate its morphology and size via its LIR motif-mediated interaction with Atg8a.

## Highlights

• Atg8a-LDS mutants accumulate autophagy substrates and have reduced lifespan
• Quantitative proteomics identifies accumulation of GMAP in Atg8a-LDS mutants
• GMAP interacts with Atg8a via a LIR motif
• Atg8a-LDS and GMAP LIR motif mutants exhibit elongated Golgi morphology

## Graphical abstract

**Figure:**
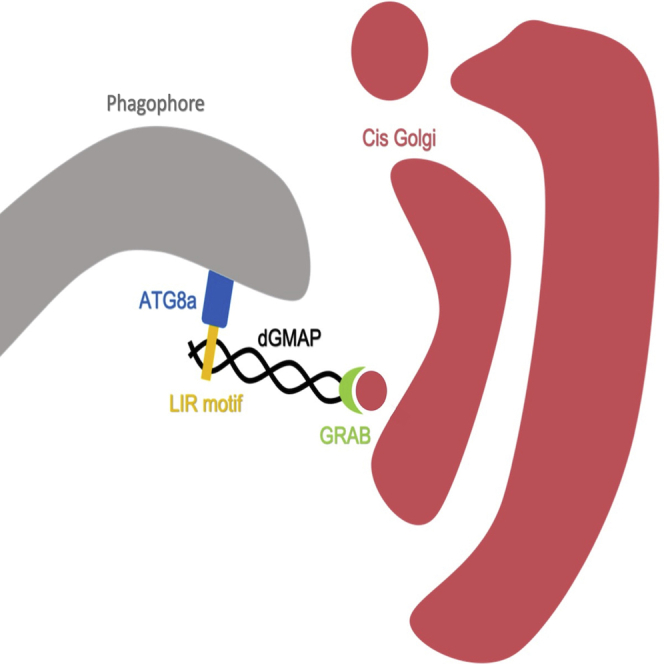

## Teaser

Rahman et al. create Atg8a-LDS (LIR motif docking site) mutants in *Drosophila*. They show that Atg8a-LDS mutants accumulate autophagy substrates and have reduced lifespan. Using quantitative proteomics, they identify Golgi protein GMAP as a LIR motif-containing protein that interacts with Atg8a. They show that GMAP LIR mutants exhibit elongated Golgi morphology.

## Introduction

Autophagy is an evolutionarily conserved process where cells degrade their own cellular material. It is involved in protein and organelle degradation and plays an essential role in both cellular and whole-animal homeostasis. Autophagy is a cellular response in nutrient starvation but also responsible for removing aggregated proteins, damaged organelles, and invading bacteria and viruses (Lamb et al., 2013; Randow and Youle, 2014). There are various types of autophagy, such as macroautophagy, microautophagy, and chaperone-mediated autophagy (Lamb et al., 2013). During macroautophagy, there is sequestration of cellular material into double-membrane vesicles called autophagosomes. The autophagosomes are subsequently fused with the lysosomes where the sequestered cargoes are degraded by lysosomal hydrolases. The products of degradation are transported back into the cytoplasm through lysosomal membrane permeases and can be reused by the cell (Lamb et al., 2013). Although it was initially believed that autophagy occurs randomly inside the cell, it is now established that sequestration and degradation of cytoplasmic material by autophagy can be selective through receptor and adapter proteins (Randow and Youle, 2014; Johansen and Lamark, 2020).

The core autophagic machinery, including Atg8 proteins, is highly conserved from yeast to humans (Johansen and Lamark, 2020). In yeast, there is a single Atg8 protein involved in selective autophagy. The evolution of multicellular organisms gave rise to two families of Atg8 proteins; MAP1LC3, often called LC3, and GABARAP. In humans the Atg8 family of proteins consists of seven proteins: LC3A, LC3B, LC3B2, LC3C, GABARAP, GABARAPL1, and GABARAPL2 (Johansen and Lamark, 2020). Atg8 proteins were discovered to be a central player in selective autophagy through their interaction with LIR motif-containing proteins. The LIR motif was initially characterized in p62 (mammals) and ATG19 (yeast) (Johansen and Lamark, 2020). Selective autophagy receptors and adapters contain short linear motifs LIR motifs (LC3-interacting region motifs), LC3 recognition sequences (LRS), or Atg8-interacting motifs (AIM), which are required for the interaction with Atg8-family proteins (Atg8/LC3/GABARAP) (Pankiv et al., 2007; Ichimura et al., 2008; Noda et al., 2010). LIR motif-containing proteins (LIRCPs) bind via their LIR motif to the hydrophobic pocket 1 (HP1) and hydrophobic pocket 2 (HP2) of the LIR docking site (LDS) of the respective Atg8 protein (Johansen and Lamark, 2020). Different variations of LIR motifs have a preference to different forms of ATG8. For example, some LIR motifs exclusively bind to GABRAP while others preferentially bind to LC3 proteins (Rogov et al., 2017; Wirth et al., 2019). In simpler multicellular organisms, such as *Drosophila* there are only two Atg8 proteins (Atg8a and Atg8b). *Drosophila* Atg8b expression is only observed in the male germline and it is required for male fertility independent of its lipidation and autophagy (Jipa et al., 2021). Sequence analysis of Atg8a is almost identical with Atg8b and suggests that Atg8a and Atg8b have LDS sites (Jipa et al., 2021). Atg8 proteins also contain a UIM (ubiquitin-interacting motif) docking site (UDS), which mediates another type of interaction that is LIR motif/LDS independent (Marshall et al., 2019). Despite the growing identification of selective autophagy receptors and adapters in mammals, the regulation and mechanisms of action of selective autophagy receptors and adapters, and the physiological significance of Atg8’s LDS docking site, are poorly described in the fruit fly *Drosophila melanogaster*.

Selective autophagy mediates the degradation of organelles (Anding and Baehrecke, 2017). However, autophagic degradation of the Golgi apparatus is not well studied (Mijaljica et al., 2006; Lu et al., 2020; De Tito et al., 2020; Nthiga et al., 2021). In this study, we created Atg8a LDS mutant flies using CRISPR. Atg8a LDS mutants exhibit a similar phenotype with Atg8a protein null mutant flies, including accumulation of ubiquitin-positive aggregates and reduced lifespan. To identify the proteins that accumulate in these mutants we performed quantitative proteomics. We identified GMAP (Golgi microtubule-associated protein), a *cis*-Golgi protein, as an Atg8a-interacting protein that regulates Golgi turnover.

## Results

### Generation and characterization of Atg8a^K48A/Y49A^ mutants

**Figure 1:**
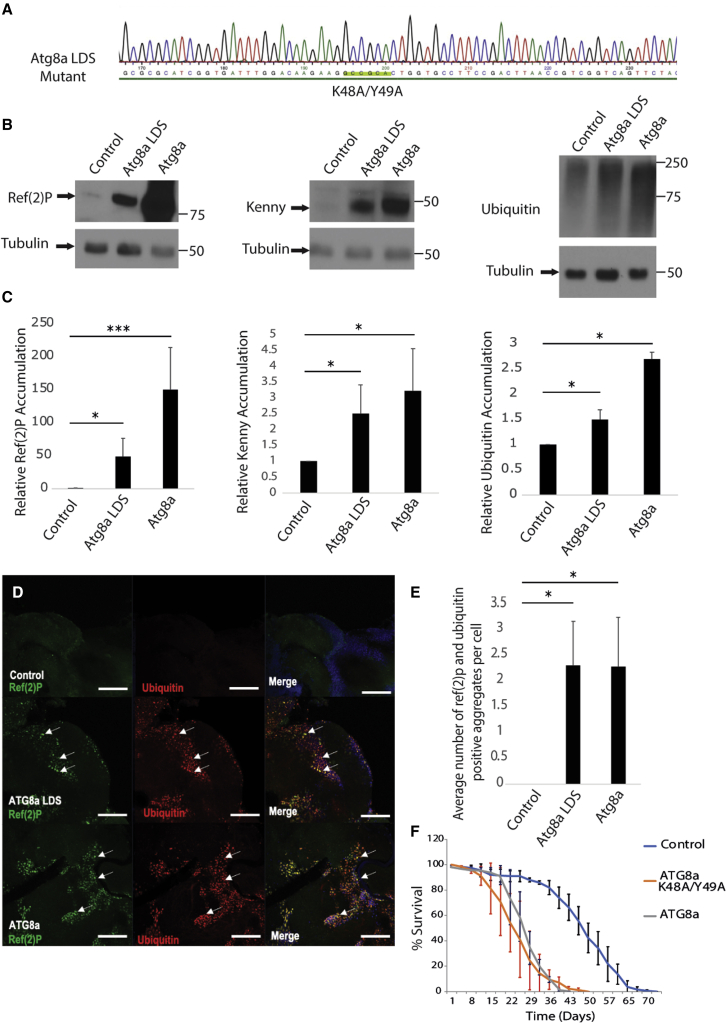
Characterization of Atg8a^K48A/Y49A^ (LDS) mutant flies (A) Genomic DNA from Atg8a ^K48A/Y49A^ mutant flies was extracted, and the sequenced results confirmed the successful incorporation of the K48A/Y49A mutation. (B) Wild-type, Atg8a^KG07569^, and Atg8a^K48A/Y49A^ mutant flies were aged for 2 weeks. Western blot analysis of lysates from whole flies showed that Ref(2)P, Kenny, and ubiquitin were accumulated in both Atg8a^KG07569^ and Atg8a^K48A/Y49A^ mutant flies. (C) Quantification of the western blottings in (B) shows significant accumulations of the aforementioned proteins in both Atg8a^KG07569^ and Atg8a^K48A/Y49A^ mutant flies. (D and E) Confocal images from 2-week-old adult brains. Ref(2)P (green) and ubiquitin (red) aggregates (arrows) can be seen in Atg8a^KG07569^ and Atg8a^K48A/Y49A^ mutant flies and not in wild-type flies. DNA was dyed with Hoechst (blue). Scale bars, 60 μm. (F) Survival test of wild-type, Atg8a ^KG07569^, and Atg8a^K48A/Y49A^ mutant flies. The results show that Atg8a and Atg8a^K48A/Y49A^ mutant flies have a short lifespan. Bar charts show means ± SD. Statistical significance was determined using two-tailed Student’s t test. ^∗^p < 0.05, ^∗∗^p < 0.01, ^∗∗∗^p < 0.001. Number of biological repeats (N = 3 for all figures). Genotypes for all figures: control: w^1118^/Y, Atg8a LDS: Atg8a ^K48A/Y49A^/Y, Atg8a: Atg8a ^KG07569^/Y.

To elucidate the physiological significance of Atg8a-LIRCPs interactions in *Drosophila*, we used CRISPR to generate Atg8a^K48A/Y49A^ (Atg8a LDS) mutants (Figure S1A). Atg8a^K48A/Y49A^ flies have two point mutations (K48A and Y49A) within the hydrophobic LDS of Atg8a that abolish interactions with LIRCPs (Johansen and Lamark, 2020). The K48A/Y49A mutation was confirmed using genomic sequencing (Figure 1A). Expression of the Atg8a protein is observed in the wild-type flies as well as the Atg8a^K48A/Y49A^ mutants (Figure S1B). This shows that Atg8a is successfully being expressed in Atg8a^K48A/Y49A^ mutant flies. Atg8a^KG07569^ mutant flies were used as a negative control as they do not express Atg8a protein (protein null mutants) (Nezis et al., 2008) (Figure S1B). Atg8a^K48A/Y49A^ flies are viable. To examine whether Atg8a^K48A/Y49A^ flies accumulate LIRCPs, we performed western blotting for Ref(2)P and Kenny, two proteins that have been shown to interact with Atg8a via LIR motifs (Jain et al., 2015; Nezis et al., 2008; Tusco et al., 2017). Western blotting analysis showed that Ref(2)P and Kenny, as well as ubiquitinated proteins, accumulated in Atg8a^K48A/Y49A^ mutant flies (Figures 1B, 1C, and S1C), indicating that the LIR motif/LDS interaction is important for their degradation by autophagy. Expression of 3xmCherry-Atg8a in Atg8a^K48A/Y49A^ mutant background was sufficient to rescue accumulation of Ref(2)P (Figure S1D). We further used immunofluorescence confocal microscopy to determine the expression of Ref(2)P in adult *Drosophila* brain and found that Atg8a^K48A/Y49A^ mutant flies showed a significant increase in the number of Ref(2)P- and ubiquitin-positive puncta (Figures 1D and 1E). In addition, we observed that Atg8a^K48A/Y49A^ mutation did not negatively affect bulk autophagy (Figures S1E and S1F). We also observed that Atg8a^K48A/Y49A^ mutant flies have a short lifespan, which was similar to that of Atg8a^KG07569^ mutant flies (Figure 1F). All together, these data show that Atg8a’s LDS docking site is physiologically important for the function of Atg8a protein to selectively degrade LIRCPs.

### Quantitative proteomics analysis of Atg8a^K48A/Y49A^ mutants

**Figure 2:**
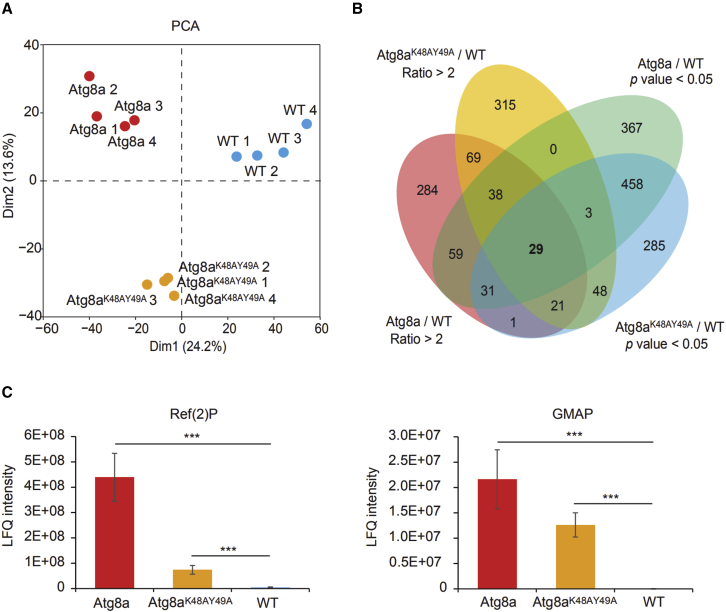
Quantitative proteomic analysis of Atg8a^K48A/Y49A^ mutant flies (A) Principal-component analysis (PCA) of wild-type, Atg8a^KG07569^, and Atg8a^K48A/Y49A^ mutant adult *Drosophila* heads. Two-week-old male flies were selected and their heads were collected to perform the proteomic analysis. Four biological replicates were performed for each sample. PCA divided the 12 protein samples into three obvious groups. (B) Venn diagram representing upregulated proteins in *Drosophila* mutant flies. The cut-off p value was set as <0.05 together with a difference of more than 2-fold between mutant and wild-type *Drosophila* heads. Twenty-nine proteins passed these two criteria and showed upregulated expression in both of Atg8a^KG07569^ and Atg8a^K48A/Y49A^ mutants. (C) The iBAQ intensity is used to show upregulation of Ref(2)P and GMAP. Bar charts show means ± SD. ^∗^p < 0.05, ^∗∗^p < 0.01, ^∗∗∗^p < 0.001. Genotypes for all figures: wild-type: w^1118^/Y, Atg8a LDS: Atg8a ^K48A/Y49A^/Y, Atg8a: Atg8a ^KG07569^/Y.

To identify the proteins that accumulate in Atg8a^K48A/Y49A^ mutants, we collected 2-week-old fly heads and performed quantitative proteomics analysis. Analysis by LC-MS/MS identified 3,036, 2,342, and 2,468 proteins from wild-type, Atg8a^KG07569^, and Atg8a^K48A/Y49A^ mutant fly heads, respectively (Table S1). Principal-component analysis divided the 12 protein samples into 3 obvious groups: wild-type, Atg8a^KG07569^, and Atg8a^K48A/Y49A^ mutant (Figure 2A). To identify the upregulated proteins in mutant flies, we set the cut-off p value as <0.05, together with a difference of more than 2-fold between mutant and wild-type flies (Table S2). Twenty-nine proteins passed these two criteria and showed upregulated expression in both Atg8a^KG07569^ and Atg8a^K48A/Y49A^ mutants (Figures 2B; Table S2). Among them, Ref(2)P (Figure 2C) has been already shown to have a functional LIR motif (Jain et al., 2015). GMAP was also shown to be significantly upregulated in Atg8a^K48A/Y49A^ flies (Figure 2C). We therefore focused to further characterize the function of GMAP in selective autophagy.

### GMAP is an Atg8a-interacting protein

**Figure 3:**
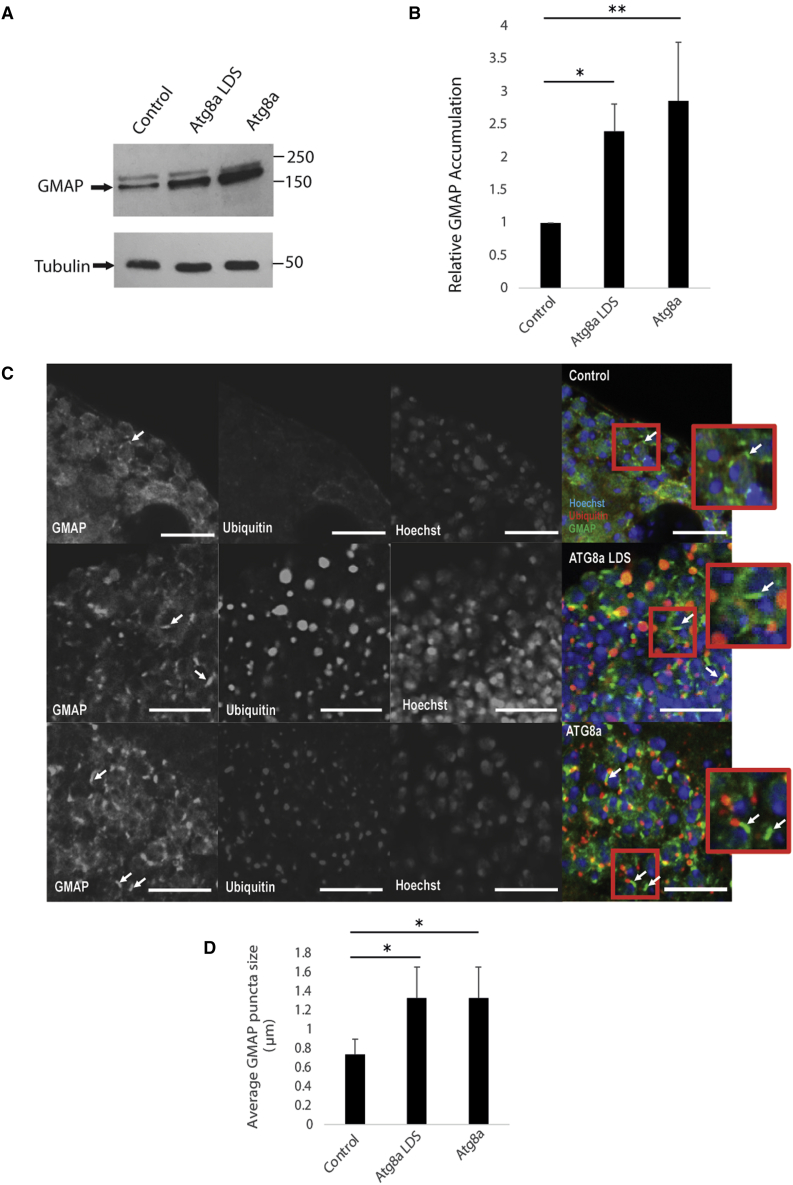
Accumulation of GMAP in Atg8a^KG07569^ and Atg8a^K48A/Y49A^ mutant flies (A) Western blot analysis shows that GMAP is accumulated in both Atg8a ^KG07569^ and Atg8a^K48A/Y49A^ mutant flies. (B) Quantification of GMAP in Atg8a^KG07569^ and Atg8a^K48A/Y49A^ mutant flies. (C) Confocal images from 2-week-old adult brains, GMAP (green) (arrows) and ubiquitin (red) aggregates can be seen in Atg8a^KG07569^ and Atg8a^K48A/Y49A^ mutant flies. DNA was dyed with Hoechst (blue). Scale bars, 10 μm. (D) Average GMAP puncta size is larger in Atg8a^KG07569^ and Atg8a^K48A/Y49A^ mutant flies compared with wild-type flies. Bar charts show means ± SD. Statistical significance was determined using two-tailed Student’s t test. ^∗^p < 0.05. Number of biological repeats (N = 3 for all figures). Genotypes for all figures: wild-type: w^1118^/Y, Atg8a LDS: Atg8a ^K48A/Y49A^/Y, Atg8a: Atg8a ^KG07569^/Y.

GMAP is a *cis*-Golgi protein that has a role in anterograde transport and Golgi organization *in vivo* (Friggi-Grelin et al., 2006; Sinka et al., 2008). To verify the proteomics data, we tested if GMAP accumulates in Atg8a LDS mutants. Western blot analysis showed that GMAP is accumulated in Atg8a^KG07569^ and Atg8a^K48A/Y49A^ mutant flies compared with wild-type flies (Figures 3A and 3B). We further used immunofluorescence confocal microscopy to determine the expression pattern of GMAP in adult *Drosophila* brain. We observed that there is a significant increase in the number of GMAP- and ubiquitin-positive structures in the adult brain of Atg8a^KG07569^ and Atg8a^K48A/Y49A^ mutant flies compared with wild-type files (Figure 3C). In addition, the size of GMAP puncta was significantly increased in Atg8a^KG07569^ and Atg8a^K48A/Y49A^ mutant flies (Figures 3C and 3D). These data suggest that selective autophagy regulates the size and morphology of the Golgi apparatus.

**Figure 4:**
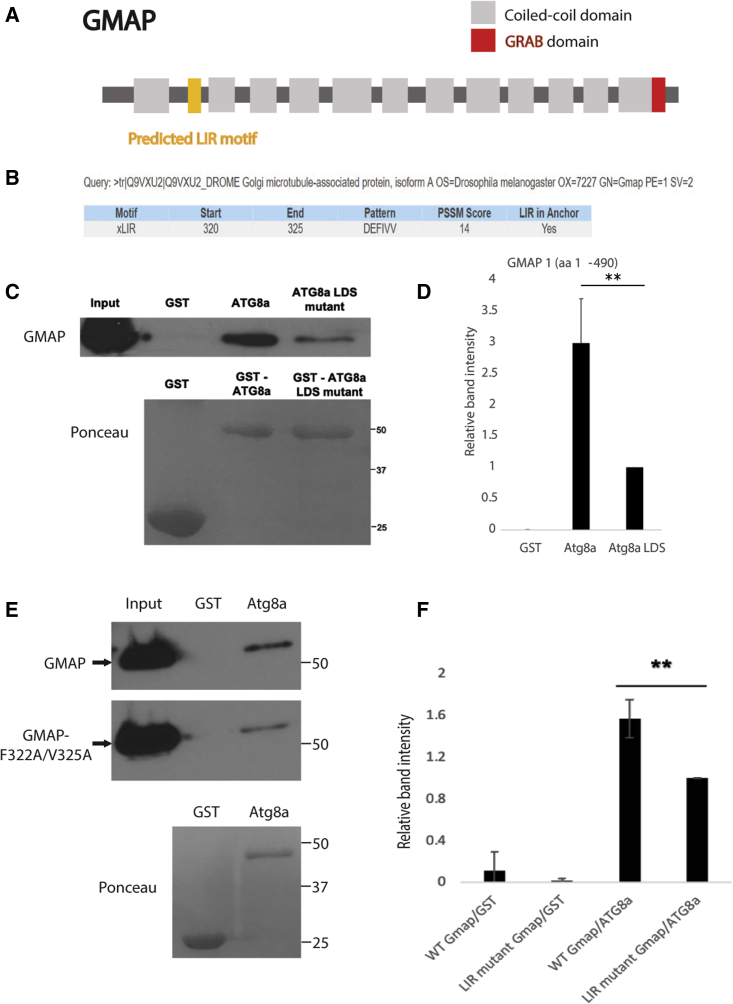
GMAP interacts with Atg8a via a LIR motif (A) Structure of GMAP. GMAP is a coiled-coil protein which has 12 coiled-coil domains (gray) and a GRAB domain (red). Yellow represents the predicted LIR motif. (B) GMAP has a predicted LIR motif at position 320–325. (C and D) GST-pulldown assay between GST-tagged Atg8a-WT or GST-tagged Atg8a-LDS mutant and His-tagged GMAP. GMAP interacts with Atg8a-WT but significantly less with Atg8a-LDS. GST was used as negative control. (E and F) GST-pulldown assay between GST-tagged Atg8a-WT and His-GMAP or His-GMAP LIR mutant. GMAP interacts with Atg8a. Point mutations of the GMAP LIR motif in positions 322 and 325 by alanine substitutions of the aromatic and hydrophobic residues (F322A and V325A) significantly reduced its binding to Atg8a. GST was used as negative control. A truncated form of GMAP (1–490 aa) was used. Bar charts show means ± SD. Statistical significance was determined using two-tailed Student’s t test. ^∗∗^p < 0.01. Number of biological repeats (N = 3 for all figures).

GMAP is a coiled-coil protein that has 12 coiled-coil domains and a GRAB domain (Friggi-Grelin et al., 2006; Sinka et al., 2008) (Figure 4A). We used iLIR software (Kalvari et al., 2014; Jacomin et al., 2016) to predict functional LIR motifs in the GMAP protein. GMAP has a predicted LIR motif at position 320–325 with the sequence DEFIVV (Figure 4B). To examine whether GMAP interacts with Atg8a and has a functional LIR motif, we performed GST pulldowns and confirmed the direct interaction between GMAP and Atg8a (Figures 4C and 4D). Atg8a-LDS showed significantly decreased binding to GMAP (Figures 4C and 4D). In addition, point mutations of the GMAP LIR motif in positions 322 and 325 by alanine substitutions of the aromatic and hydrophobic residues (F322A and V325A) reduced its binding to Atg8a (Figures 4E and 4F). These results show that GMAP interacts with Atg8a and that LIR motif at position 320–325 is important for this interaction.

### GMAP mediates Golgi turnover

**Figure 5:**
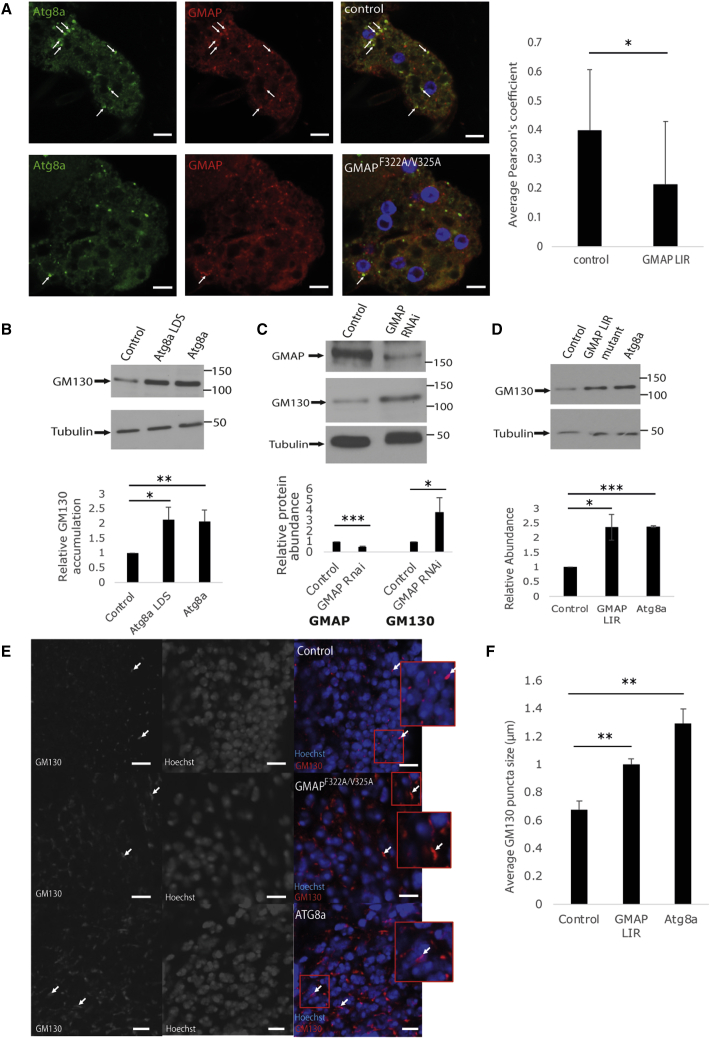
GMAP regulates Golgi turnover via autophagy (A) Confocal images showing co-localization of endogenous Atg8a and the GMAP under starvation conditions in adult fat body in control and GMAP ^F322A/V325A^ mutant flies. (B) Western blots showing accumulation of the Golgi marker GM130 in Atg8a ^KG07569^ and Atg8a^K48A/Y49A^ mutant flies compared with wild-type flies. (C) Western blots showing accumulation of GM130 in GMAP-RNAi lines compared with control RNAi and its quantification shown below. (D) Western blots showing accumulation of GM130 in GMAP ^F322A/V325A^ mutant flies compared with wild-type flies. (E) Immunofluorescence confocal microscopy of *Drosophila* brain showing increased accumulation of the *cis*-Golgi marker (GM130) and the altered morphology of Golgi in GMAP ^F322A/V325A^ and Atg8a ^KG07569^ mutant flies. Scale bars, 10 μm. (F) Average GM130 puncta size is larger in GMAP ^F322A/V325A^ and Atg8a ^KG07569^ mutant flies compared with wild-type flies. Bar charts show means ± SD. Statistical significance was determined using two-tailed Student’s t test. ^∗^p < 0.05, ^∗∗^p < 0.01, ^∗∗∗^p < 0.001. Number of biological repeats (N = 3 for all figures). Genotypes: (A) Control: w^1118^/Y, GMAP: GMAP ^F322A/V325A^/Y. (B) Control: w^1118^/Y, Atg8a LDS: Atg8a ^K48A/Y49A^/Y, Atg8a: Atg8a ^KG07569^/Y. (C) Control: yw^1118^;P{attP,y[+],w[3`]}/+;da-GAL4/+, GMAP-RNAi: GMAP-RNAi/+; da-GAL4/+. (D and E) Control:w^1118^/Y, GMAP: GMAP ^F322A/V325A^/Y, Atg8a: Atg8a ^KG07569^/Y.

Since GMAP is a Golgi protein that interacts with Atg8a we examined if it regulates the autophagic degradation of the Golgi complex. We observed that GMAP colocalizes with Atg8a during starvation (Figure 5A). We also observed that the Golgi marker GM130 accumulates in Atg8a^K48A/Y49A^ and Atg8a^KG07569^ mutants, suggesting that autophagy regulates Golgi turnover (Figure 5B). In addition, knockdown of GMAP led to accumulation of GM130 (Figure 5C). To elucidate the role of GMAP in Golgiphagy we used CRISPR-Cas9 technology to generate GMAP LIR mutants (GMAP F322A V325A) (Figures S2A). GMAP^F322A/V325A^ mutants are homozygous viable. GMAP colocalization with Atg8a during starvation is significantly decreased in GMAP^F322A/V325A^ mutants (Figure 5A). To examine whether GMAP^F322A/V325A^ mutant flies accumulate Golgi markers, we used western blotting. We observed that GM130 significantly accumulates in GMAP LIR mutant flies (Figure 5D). We further used immunofluorescence confocal microscopy to examine the morphology of the Golgi of GMAP^F322A/V325A^ and Atg8a ^KG07569^ flies using GM130 immunostaining. We observed that Golgi appeared to be deformed and elongated compared with wild-type flies (Figures 5E, 5F, and S2B). To further examine Golgi morphology, we used transmission electron microscopy.

We observed that the area and length of Golgi compartments is significantly larger in GMAP^F322A/V325A^ mutants compared with controls (Figure S3). All together, these results suggest that GMAP regulates Golgi complex turnover via selective autophagy.

To examine the role of Atg8aK48A/Y49A and GMAPF322A/V325A mutation in Golgi complex function, we monitored the release of the Glue-Red reporter from the salivary glands of late L3 larvae (Costantino et al., 2008; Csizmadia et al., 2018). As shown in Figure S4, we did not observe secretory defects (Figure S4). To confirm these results, we also investigated the possible retention of the collagen reporter Vkg:GFP (Morin et al., 2001) and the secr-GFP reporter (Pfeiffer et al., 2000), both of which have been used previously to show defects in the secretory machinery (Ke et al., 2018; Zang et al., 2015). Similarly, we found no notable secretion defects (Figure S5).

## Discussion

Molecular mechanisms of selective autophagy are mostly characterized in mammals (Johansen and Lamark, 2020). To investigate selective autophagy in the fruit fly *Drosophila melanogaster*, we created Atg8a LDS mutants that cannot bind to LIRCPs. Atg8a LDS mutants have a similar phenotype with Atg8a^KG07569^ mutants (that do not express Atg8a protein): (1) they are viable, (2) they accumulate experimentally verified LIRCPs and ubiquitinated proteins, and (3) and they have reduced lifespan. These results show that LIR motif/LDS interaction is important for the function of Atg8a in autophagy *in vivo*. The accumulation of LIRCPs and ubiquitinated proteins is milder in Atg8a LDS mutants compared with Atg8a^KG07569^ mutants. We speculate that this could be related to a UIM docking site (UDS) that it is not mutated in Atg8a LDS mutants and could contribute to degradation of autophagic substrates. The moderate increase in bulk autophagy observed in Atg8a LDS mutants could be related to stabilization of Atg8a LDS mutant protein, since it is not consumed by LDS binding proteins being degraded by autophagy (like Ref(2)P).

Selective autophagy of mitochondria, peroxisomes, lysosomes, and ER has been described and their receptors have been identified (Anding and Baehrecke, 2017; Johansen and Lamark, 2020). Golgi turnover by autophagy is poorly described. Recently, work by the Johansen group identified CALCOCO1 as a selective autophagy receptor for Golgiphagy (Nthiga et al., 2021). They showed that CALCOCO1 binds the Golgi palmitoyl-transferase ZDHHC17 to mediate Golgi degradation by autophagy during starvation. Depletion of CALCOCO1 causes expansion of the Golgi and accumulation of its proteins. Here, we show that, in *Drosophila*, *cis*-Golgi protein GMAP binds directly to Atg8a (without the involvement of an intermediate receptor) to mediate Golgi turnover and control the size and morphology of the Golgi complex. GMAP binding to Atg8a is mediated by a LIR motif. The observation that interaction of GMAP with Atg8a is not completely abolished by Atg8a^K48A/Y49A^ or GMAP^F322A/V325A^ mutations could suggest the presence of additional LIR motifs or binding domains.

GMAP^F322A/V325A^ mutants exhibit accumulation of *cis*-Golgi markers and elongated Golgi morphology, suggesting a role of GMAP in Golgi turnover. The observations that Atg8a^K48A/Y49A^ or GMAP^F322A/V325A^ mutations did not affect secretion of known secreted proteins suggest that Atg8a and GMAP’s LIR motif are not absolutely essential for Golgi secretory function and there may be a redundancy with other proteins (yet to be identified) in the regulation of Golgi complex turnover in relation to its secretory function.

In summary, we have shown that the LDS binding pocket in Atg8a plays an important role in the execution of selective autophagy. We identified the *cis*-Golgi protein GMAP as an Atg8a-interacting protein. We suggest that GMAP mediates Golgi turnover via its LIR motif-mediated interaction with Atg8a. Our study highlights the physiological importance of Atg8a’s LDS binding pocket and opens new avenues in the regulation of Golgi turnover by selective autophagy.

### Limitations of the study

GMAP was identified as an Atg8a-interacting protein in a proteomics screening in Atg8a LDS mutants. We also identified several other proteins that accumulate in Atg8a LDS mutants. However, our proteomics analysis might not have identified all accumulating proteins. In addition, this study does not characterize other putative docking sites in Atg8a protein, such as the UDS.

## STAR★Methods

### Key resources table

**Table:** 

REAGENT or RESOURCE	SOURCE	IDENTIFIER
**Antibodies**		

Rabbit anti-Ref(2)P	Abcam	Cat#ab178440
anti-Kenny	Gift from Dr N. Silverman	N/A
Mouse anti-mono/poly-ubiquitinated proteins (FK2)	Enzo	Cat#BML-PW8810-0100
Rabbit anti-GM130	Abcam	Cat#ab30637
Mouse anti-6xHis tag®	Abcam	Cat#ab18184
Rabbit anti-dGMAP (Western blotting)	Gift from Dr Pascal Therond	N/A
Goat anti-GMAP (IF)	Developmental Studies Hybridoma Bank	RRID:AB_2618259
Mouse anti-alpha tubulin	Sigma-Aldrich	Cat#T5168; RRID:AB_477579
Rabbit anti-Gabarap+GabarapL1+GabarapL2	Abcam	Cat#ab109364
Rabbit Anti-Mouse IgG HRP	Thermo Fisher Scientific	Cat#31450; RRID:AB_228427
Goat Anti-Rabbit IgG HRP	Thermo Fisher Scientific	Cat#31460; RRID:AB_228341
Rabbit Anti-Goat IgG CF488A	Sigma-Aldrich	Cat#SAB4600053
Goat Anti-Mouse IgG CF488A	Sigma-Aldrich	Cat#SAB4600042
Chicken Anti-Goat IgG CF488A	Sigma-Aldrich	Cat#SAB4600028
Goat Anti-Rabbit IgG CF568	Sigma-Aldrich	Cat#SAB4600084
Goat Anti-Mouse IgG CF568	Sigma-Aldrich	Cat#SAB4600082
Donkey Anti-Mouse IgG CF568	Sigma-Aldrich	Cat#SAB4600075

**Bacterial and virus strains**		

Rosetta™ 2(DE3) Singles™ Competent Cells	Novagen	Cat#71400

**Chemicals, peptides, and recombinant proteins**		

cOmplete™ ULTRA Tablets, Mini, EDTA-free, *EASYpack* Protease Inhibitor Cocktail	Roche	Cat#5892791001
Glutathione Sepharose® 4B	Sigma-Alrich	Cat#17-0756-01
Formaldehyde	Sigma-Alrich	Cat#F8775

**Critical commercial assays**		

QuickChange site-directed mutagenesis	Stratagene	200523
BigDye™ Terminator v3.1 Cycle Sequencing Kit	Applied Biosystems	Cat#4337455
Phusion™ High-Fidelity DNA Polymerase	Thermo Fisher Scientific	Cat#F-530XL

**Deposited data**		

Identified proteins from Atg8aKG07569 mutants, Atg8aK48A/Y49A mutants, and wild-type (WT) flies	This paper	Table S1
Differential protein expression between WT and mutant Drosophila	This paper	Table S2

**Experimental models: Organisms/strains**		

*Drosophila melanogaster*: control/WT: w^1118^/Y	Bloomington *Drosophila* stock center	#3605
*Drosophila melanogaster*: da-GAL4: w[^∗^]; P{w[+mW.hs]=GAL4-da.G32}UH1, Sb[1]/TM6B, Tb[1]	Bloomington *Drosophila* stock center	#55851
*Drosophila melanogaster*: Atg8a LDS: Atg8a^K48A/Y49A^/Y	This paper; created by CRISPR-mediated mutagenesis; Well Genetics	N/A
*Drosophila melanogaster*: Atg8a: Atg8a^KG07569^/Y	Nezis et al. (2008)	N/A
*Drosophila melanogaster*: Control RNAi: y,w^1118^;P{attP,y[+],w[3`]}/+;da-GAL4/+	Vienna *Drosophila* Resource Center	#60100
*Drosophila melanogaster*: GMAP-RNAi: P{KK107249}VIE-260B	Vienna *Drosophila* Resource Center	#108063
*Drosophila melanogaster*: GMAP LIR: GMAP^F322A/V325A^/Y	This paper; created by CRISPR-mediated mutagenesis; Well Genetics	N/A
*Drosophila melanogaster*: secr.EGFP: UAS-secr.EGFP/+; tub-GAL4/+	Gift from Dr Jean-Paul Vincent	N/A
*Drosophila melanogaster*: Vkg::GFP: P{PTT-un}vkg^G00454^/+	Gift from Prof József Mihály	N/A
*Drosophila melanogaster*: P{Sgs3-DsRed}	Gift from Andrew Andres	N/A

**Oligonucleotides**		

GMAP truncated protein: Forward Primer: CGGAATTCATGTCGTGGCTGAACAGC	This paper	N/A
GMAP truncated protein: Reverse Primer (R490): CCGCTCGAGTTATTAGTCCGCATCGTCCA	This paper	N/A
Primers for GMAP F322A/V325A mutagenesis: Forward GCACAGCGAGGATGAGGCCATAG TTGCACGCCAAGCGGATGCC	This paper	N/A
Primers for GMAP F322A/V325A mutagenesis: Reverse Primer: GGCATCCGCTTGGCGTGCAACT ATGGCCTCATCCTCGCTGTGC	This paper	N/A

**Recombinant DNA**		

Plasmid: pET28a(+)	Novagen	Cat#69864

**Software and algorithms**		

iLIR database	Kalvari et al. (2014)	http://repeat.biol.ucy.ac.cy/iLIR/
iTEM software	N/A	https://www.itemsoft.com/

### Resource availability

#### Lead contact

Additional information and requests for reagents and protocols should be directed to and will be fulfilled by the Lead Contact, Ioannis Nezis (I.Nezis@warwick.ac.uk).

#### Materials availability

All materials are publicly available. Please contact Prof. Ioannis Nezis.

### Experimental model and subject details

#### Fly husbandry and generation of transgenic lines

Flies used in experiments were kept at 25°C and 70% humidity raised on cornmeal-based feed. Atg8a [Atg8a^KG07569^] flies were from our lab (Nezis et al., 2008). da-GAL4 (#55851) and w^1118^ control/WT (#3605) flies were obtained from the Bloomington Drosophila stock centre. GMAP-RNAi (#108063) and control RNAi (#60100) flies were obtained from the Vienna Drosophila Resource Center. secr.EGFP flies were gifted by Dr Jean-Paul Vincent and Vkg::GFP flies were gifted by Prof József Mihály. Sgs3-dsRed2 (or simply GlueRed, controlled by genomic *sgs3* promoter) reporter was kindly provided by Andrew Andres, University of Nevada, Las Vegas, NV, USA (Costantino et al., 2008).

CRISPR-mediated mutagenesis to create Atg8a^K48A/Y49A^ and GMAP^F322A/V325A^ mutants was performed by WellGenetics Inc. using modified methods of Kondo and Ueda (2013). In brief, gRNA sequences CGGTCAGGTCGGAAGGCACC[AGG] (for Atg8a) and GTTCATAGTTGTACGCCAAG[CGG] (for GMAP) were cloned into U6 promoter plasmid(s). Cassette K48A/Y49A-PBacDsRed or F322A/V325A-PBacDsRed containing two PiggyBac sites, 3xP3-DsRed, designed point mutation and two 1kb-homology arms were cloned into pUC57-Kan as donor template for repair. The two homology arms of Atg8a/CG32672 or Gmap/CG33206 were amplified by Phusion High-Fidelity DNA Polymerase (Thermo Scientific) from genomic DNA at optimized condition, which reflects sequences of the injection strain *in vivo*. Any mismatches found in coding regions are considered as polymorphisms. The K48A/Y49A-PBacDsRed or F322A/V325A-PBacDsRed cassette was obtained from sequence verified plasmid stock by restriction enzyme digestion. The cassette and two homology arms were cloned by sequence ligation-independent cloning method into vector pUC57-Kan, followed by standard transformation protocol, colony PCR selection and sequencing. Two homology arms and junctions of cassette fragment(s) were confirmed by PCR and sequencing. Atg8a-targeting or GMAP-targeting gRNAs and hs-Cas9 were supplied in DNA plasmids, together with donor plasmid for microinjection into embryos of control strain w1118. F1 flies carrying selection marker of 3xP3-RFP were further validated by genomic PCR and sequencing. CRISPR generates a break in Atg8a or GMAP and is replaced by cassette K48A/Y49A-PBacDsRed or F322A/V325A-PBacDsRed respectively. For ATG8a K48A Y49A CRISPR mutants, 13 putative mutants tested and finally 4 independent lines were established. For GMAP F322A V325A CRISPR mutants, 6 putative mutants tested and finally 1 independent line was established.

### Method details

#### Protein extraction and western blotting

Protein content was extracted from the head and the full fly body in RIPA lysis buffer (50 mM Tris pH 7.4, 150 mM NaCl, 1% Igepal, 0.5% sodium deoxycholate, 0.1% SDS supplemented with cOmplete™ ULTRA EDTA-free protease inhibitor cocktail (Roche, #5892791001)) using a motorized mortar and pestle. Protein concentrations were determined by the Bradford method. 100–200 μg proteins were loaded on polyacrylamide gels and were transferred onto PVDF membranes (cold wet transfer in 10% ethanol for 1 h at 100 V). Membranes were blocked in 5% non-fat milk in TBST (0.1% Tween-20 in TBS) for 1 h. Primary antibodies diluted in TBST were incubated overnight at 4C with gentle agitation. HRP-coupled secondary antibodies binding was done at room temperature (RT) for 1 h in 1% non-fat milk dissolved in TBST and ECL mix incubation for 2 min. All washes were performed for 10 min in TBST at RT.

#### Immunohistochemistry

Fly tissues were dissected in PBS and fixed for 30 min in 4% formaldehyde (#F8775) in PBS. Blocking and antibody incubations were performed in PBT (0.3% BSA, 0.3% Triton X-100 in PBS). Primary antibodies were incubated overnight at 4°C in PBST, secondary antibodies were incubated 2 h at room temperature in PBST. Samples were observed under a Zeiss 880 confocal microscope and an ApoTome 2-fitted Zeiss AxioImager M.2 microscope.

#### Proteomics

Proteins were extracted from the drosophila head by using RIPA buffer. Solubilized proteins were recovered by centrifugation (12,000 g, 10 min) and placed in an ultrafiltration tube (MWCO 3,000, Millipore, USA), and reduced with 15 mM dithiothreitol for 120 min, and alkylated with 50 mM iodoacetamide for 60 min in the dark. Protein samples were washed three times with 50 mM NH4HCO3 and then digested with trypsin at a weight ratio of 1:50 (trypsin:protein) for 20 h at 37°C. Tryptic peptides were recovered by centrifugation, lyophilized, and resuspended in 40 μL 0.1% formic acid. Tryptic peptides (4 μL) were separated on a Thermo Fisher Scientific EASY-nLC 1000 system using a Thermo Fisher Scientific EASY-Spray column (C18, 2 μm, 100 Å, 50 μm × 15 cm), and were analyzed using a Thermo Scientific Q Exactive mass spectrometer. Four biological replicates were used for the LC-MS/MS analyses. Mass spectra raw data were analysed using MaxQuant software. Peptide searches were performed with Andromeda search algorithms. All common contaminants and reverse hits were removed. The label-free intensity quantification (LFQ) algorithm in MaxQuant was used to estimates the protein abundance. Identified proteins were listed in Table S1. FactorMineR was used to perform PCA analysis on the basis of protein expression (log10-transformed LFQ values). To find the differential proteins between wild-type and mutant drosophila, unpaired *t*-tests were used with a significance level set at p < 0.05 and a cut-off difference of more than two-fold (Table S2). LIR motifs were predicted using iLIR software at https://ilir.warwick.ac.uk.

#### Lifespan measurement

Fly lines used were isogenic. Atg8a LDS (Atg8a^K48A/Y49A^) and Atg8a (Atg8a^KG07569^, (Scott et al., 2007)) mutants were backcrossed to the w^1118^ strain for at least 6 generations to produce isogenic lines. We used the Kaplan-Meier method to measure lifespan of flies, which estimates survival probability of each risk group according to daily death events counted. Male and female flies were collected within 24 h from hatching and cohorts of 20–25 flies were maintained on standard Drosophila food at 25°C in a humidified incubator. Flies were transferred into new tubes every 2–3 days. Dead events were recorded daily. Atg8a LDS mutants eclose at the same frequency as background matched controls. Survival curves were constructed in Prism (GraphPad, versions 8 and 9), which was also used to perform the statistical analysis for curve comparison using the Mantel-Cox test.

#### Plasmid constructs

GMAP plasmids were obtained from Drosophila Genomics Resource Centre. Sequences of the GMAP were amplified by PCR and inserted in desired plasmid using either Gateway recombination system or restriction enzyme cloning. PCR products were amplified from cDNA using Phusion high fidelity DNA polymerase with primers containing the Gateway recombination site or restriction enzyme sites for Gateway entry vector and cloned into pDONR221 or pENTR using Gateway recombination cloning. Point mutants were generated using the QuickChange site-directed mutagenesis (Stratagene, #200523). Plasmid constructs were verified by conventional restriction enzyme digestion and/or by DNA sequencing (Applied Biosystems, #4337455).

#### GST pull-down assays

Bait proteins (GST alone and GST-Atg8a) as well as prey proteins (GMAP and GMAP LIR mutants) were expressed in RosettaTM 2(DE3) competent cells (Novagen, #71400). GST pull-down assays were performed using recombinant proteins produced in bacteria. Bait proteins were captured using Glutathione-Sepharose beads (Glutathione Sepharose® 4B (Sigma-Aldrich, #17-0756-01)), which were incubated for 40 mins at 4°C. A volume of 10 mL of the *in vitro* translation reaction products (0.5 mg of plasmid in a 25 mL reaction volume) were incubated with 1–10 mg of GST-recombinant protein in 200 mL of NETN buffer (50 mM Tris, pH 8.0, 150 mM NaCl, 1 mM EDTA, 0.5% Nonidet P-40, 1 mM dithiothreitol supplemented with cOmplete™ ULTRA Mini EDTA-free protease inhibitor cocktail (Roche, #5892791001) for 2 h at 4°C, washed six times with 1 mL of NETN buffer, boiled with 2X SDS gel loading buffer, and subjected to SDS-PAGE. Gels were stained with Coomassie Blue and vacuum-dried.

#### Transmission electron microscopy

Brains of 20 days (after emerging from the pupal case) old adult control and mutant animals were dissected in ice cold PBS, then fixed with 3.2% paraformaldehyde, 1% glutaraldehyde, 1% sucrose, and 3 mM CaCl_2_ in 0.1 N sodium cacodylate (pH 7.4, overnight, 4°C). Next day samples were washed with sodium cacodylate then post-fixed in 0.5% osmium tetroxide (60 min, RT) then in half-saturated aqueous uranyl acetate (30 min, RT). Samples were then dehydrated in graded series of ethanol and embedded in araldite to the manufacturer’s instructions. Ultrathin sections (from 5 control and 5 mutant animals) were stained with Reynold’s lead citrate and viewed at 80 kV operating voltage on a JEM-1011 transmission electron microscope (JEOL) equipped with a Morada digital camera (Olympus) using iTEM software (Olympus). All reagents and materials used for electron microscopy were obtained from Merck. The area and width of Golgi apparatuses of cortical neurons of the protocerebrum were measured using iTEM software (Olympus).

### Quantification and statistical analysis

#### Quantification of transmission electron microscopy

5 individuals per genotype were analysed (total 10 individuals). Neurons originated from the cortex of the protocerebrum were analysed. Out of hundreds of cells examined, 27 control and 28 GMAP LIR mutant cells contained one "whole" Golgi. The area and width of Golgi apparatuses were measured using iTEM software (Olympus). Cells containing at least three Golgi cisterns were quantified only. Cells in which the Golgi apparatus contained less than three cisterns or contained only vesicles were not evaluated as these Golgi apparatuses were considered partial. The quantified data were evaluated using SPSS21 (IBM) and independent samples u-test.

#### Quantification of western blots

Statistical analyses were done with Prism6/7 software (GraphPad). Western blot protein bands were quantified using ImageJ/FIJI 2.0 using the Gels tool. A histogram was generated for each band where the peaks were proportional to the intensity of the band. The area under the curve was used as the quantitative value. Where necessary these bands were normalised to control bands. At least three biological repeats were done and averaged. For the comparison of two groups, a two-tailed *t*-test was used.

#### Quantification of immunohistochemistry

Quantifications in Figures 1D, 1E, 3C, D, 5E, 5F and S2B: For every figure the data are from 5 images per brain, 5 brains per genotype (25 images per genotype). 100 puncta per image were assessed. The measurement of puncta size was done with the line tool in ImageJ/FIJI 2.0.

Quantifications in Figure 5A: wild type:13 fat bodies (1 per fly), 20 images. GMAP LIR: 12 fat bodies (1 per fly), 14 images. Signal colocalization was assessed by Pearson’s correlation co-efficient within the set regions of interest (ROIs) which were obtained through the creation of binary masks and background subtracted for the creation of ROIs where appropriate. Counts and puncta size were obtained similarly through the creation of binary masks and the Analyze Particles tool utilised, and counts normalised to the confocal image area in μm^2^. Compound figures were assembled in Adobe Illustrator 2022 (version 26.2).

## Data Availability

• All data generated and reported in this paper are available from the lead contact upon request. • This paper does not report original code. • Any additional information required to reanalyse the data reported in this paper is available from the lead contact upon request

## Supplemental information

Document S1. Figures S1–S5 and Table S2

Table S1. List of proteins identified in Atg8aKG07569, Atg8aK48A/Y49A, and wild-type (WT) flies using quantitative proteomics, related to Figure 2

Document S2. Article plus supplemental information
