# Supplementary material for: GMAP is an Atg8a-interacting protein that regulates Golgi turnover in Drosophila
Source: Cell Rep. 2022 May 31;39(9):110903. doi: 10.1016/j.celrep.2022.110903 (PMC9637997; doi:10.1016/j.celrep.2022.110903)
Supplement: Document S1. Figures S1–S5 and Table S2 [file mmc1.pdf]

Cell Reports, Volume *39*

Supplemental information

GMAP is an Atg8a-interacting protein

that regulates Golgi turnover in *Drosophila*

Ashrafur Rahman, Peter Lőrincz, Raksha Gohel, Anikó Nagy, Gábor Csordás, Yan Zhang, Gábor Juhász, and Ioannis P. Nezis

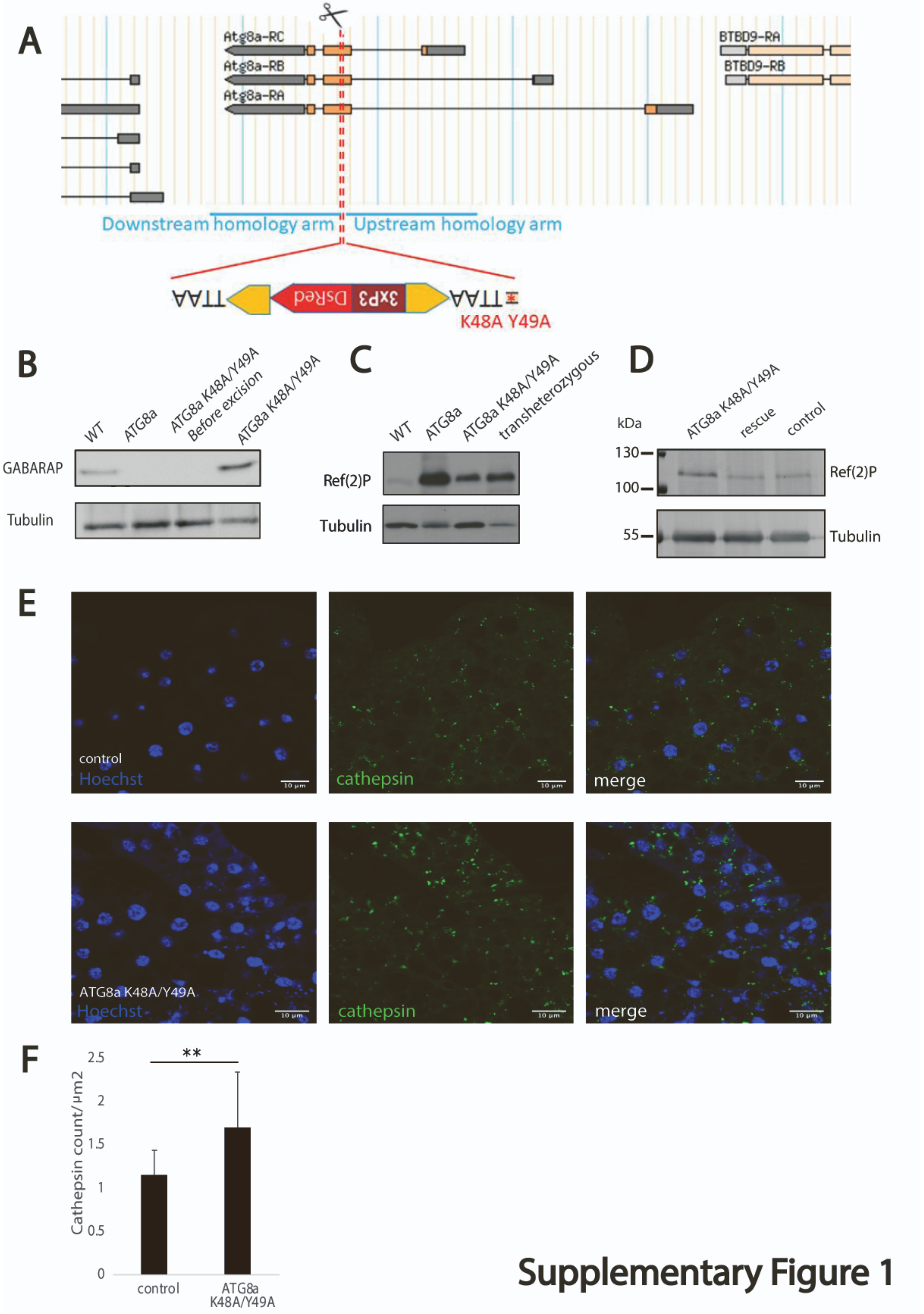

Supplementary Figure 1

Supplementary Figure 1 (Related to Figure 1). Characterization of Atg8a$^{K48A/Y49A}$ mutants.

(A) Schematics showing the generation of Atg8a$^{K48A/Y49A}$ mutant flies using CRISPR. **(B)** Western blots showing expression of Atg8a. Expression of Atg8a protein can be seen in the wild type flies (WT) as well as the Atg8a$^{K48A/Y49A}$ mutants. This shows Atg8a is successfully being expressed in the Atg8a CRISPR mutant flies. Atg8a ^{KG07569} flies were used as a negative control as there was no expression of Atg8a. There is also no expression of Atg8a in the Exon2 line prior to excision which is expected as the selectable marker must first be excised before proper expression can take place. **(C)** Western blots showing Ref(2)P accumulation in Atg8a ^{KG07569}, Atg8a$^{K48A/Y49A}$ and Atg8a ^{KG07569}/Atg8a$^{K48A/Y49A}$ trans-heterozygous flies. **(D)** Western blot showing rescue of Ref(2)P accumulation in Atg8a$^{K48A/Y49A}$ flies with co-expression of 3xmCherry-Atg8a. **(E-F)** Cathepsin L staining of adult fat body in starved wild type and Atg8a$^{K48A/Y49A}$ mutant flies. Number of biological repeats (N)=3 for all figures.

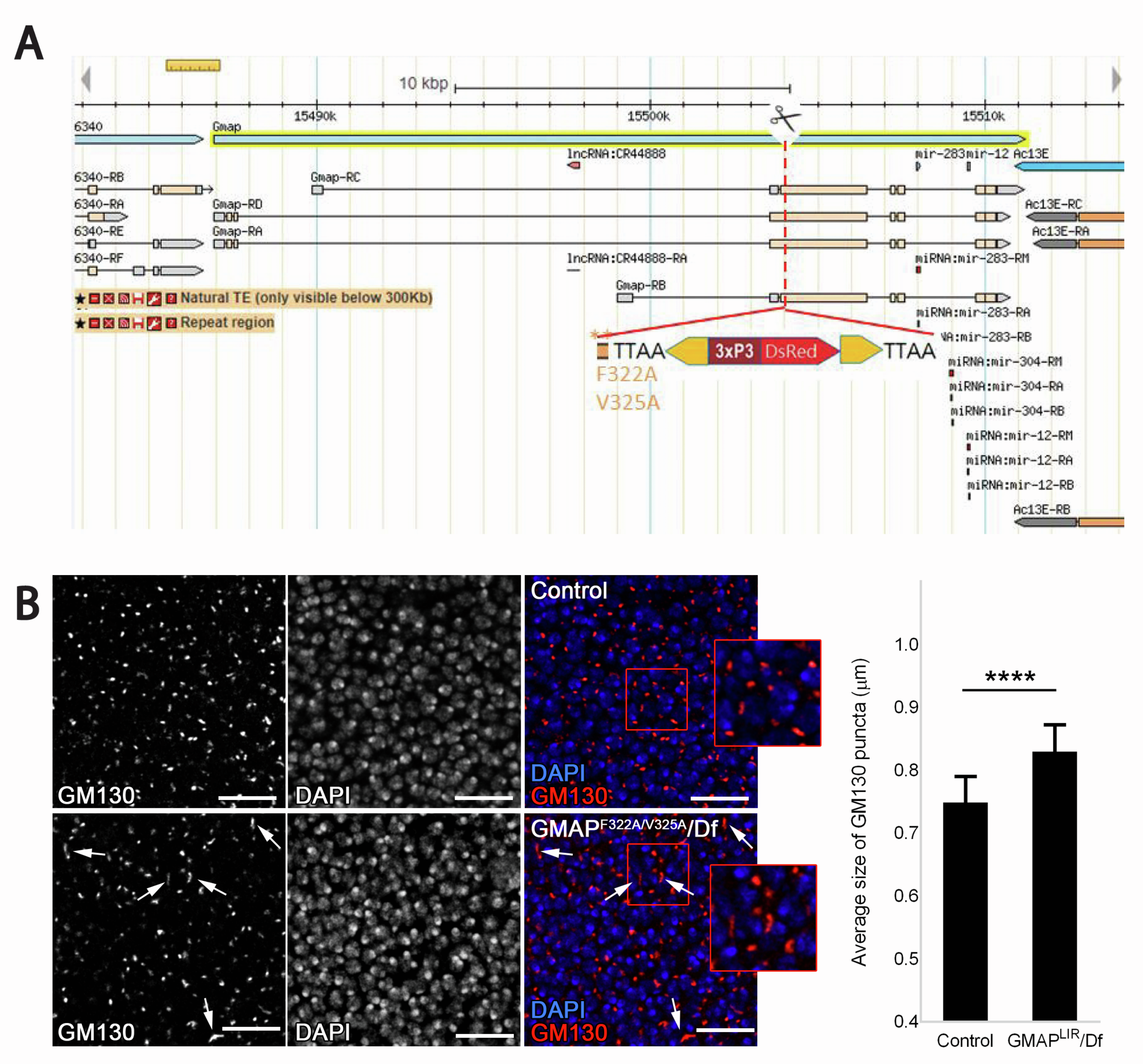
A
10 kbp
15490k
15500k
15510k
6340
Gmap
lncRNA:CR44888
mir-283
mir-12
Ac13E
6340-RB
Gmap-RC
6340-RA
Gmap-RD
Ac13E-RC
6340-RE
Gmap-RA
Ac13E-RA
6340-RF
lncRNA:CR44888-RA
miRNA:mir-283-RM
Gmap-RB
Natural TE (only visible below 300Kb)
Repeat region
miRNA:mir-283-RA
TTAA
3xP3
DsRed
TTAA
F322A
V325A
miRNA:mir-304-RM
miRNA:mir-304-RA
miRNA:mir-304-RB
miRNA:mir-12-RM
miRNA:mir-12-RA
miRNA:mir-12-RB
Ac13E-RB
B
GM130
DAPI
Control
DAPI
GM130
GM130
DAPI
GMAPF322A/V325A/Df
DAPI
GM130
Average size of GM130 puncta (μm)
1.0
0.9
0.8
0.7
0.6
0.5
0.4
****
Control
GMAPLIR/Df

Supplementary Figure 2 (Related to Figure 5). GMAP$^{F322A/V325A}$ mutants exhibit elongated Golgi morphology.

(A) Schematics showing the generation of GMAP$^{F322A/V325A}$ mutant flies using CRISPR. (**B**) Similarly to GMAP$^{F322A/V325A}$ homo/hemizygotes, GMAP $^{F322A/V325A}$ /Df trans-heterozygotes show increased Golgi marker (GM130) accumulation and altered Golgi morphology. Images are single confocal planes. Quantification of average size of GM130 puncta. Average GM130 puncta size is larger in GMAP $^{F322A/V325A}$ /Df brains was significantly higher than in wild type flies. Bar charts show means ± s.d. Statistical significance was determined using two-tailed Student's *t*-test. ****$p < 0.0001$. Number of biological repeats (N)=3 for all figures. Scale bars: 10 μm.

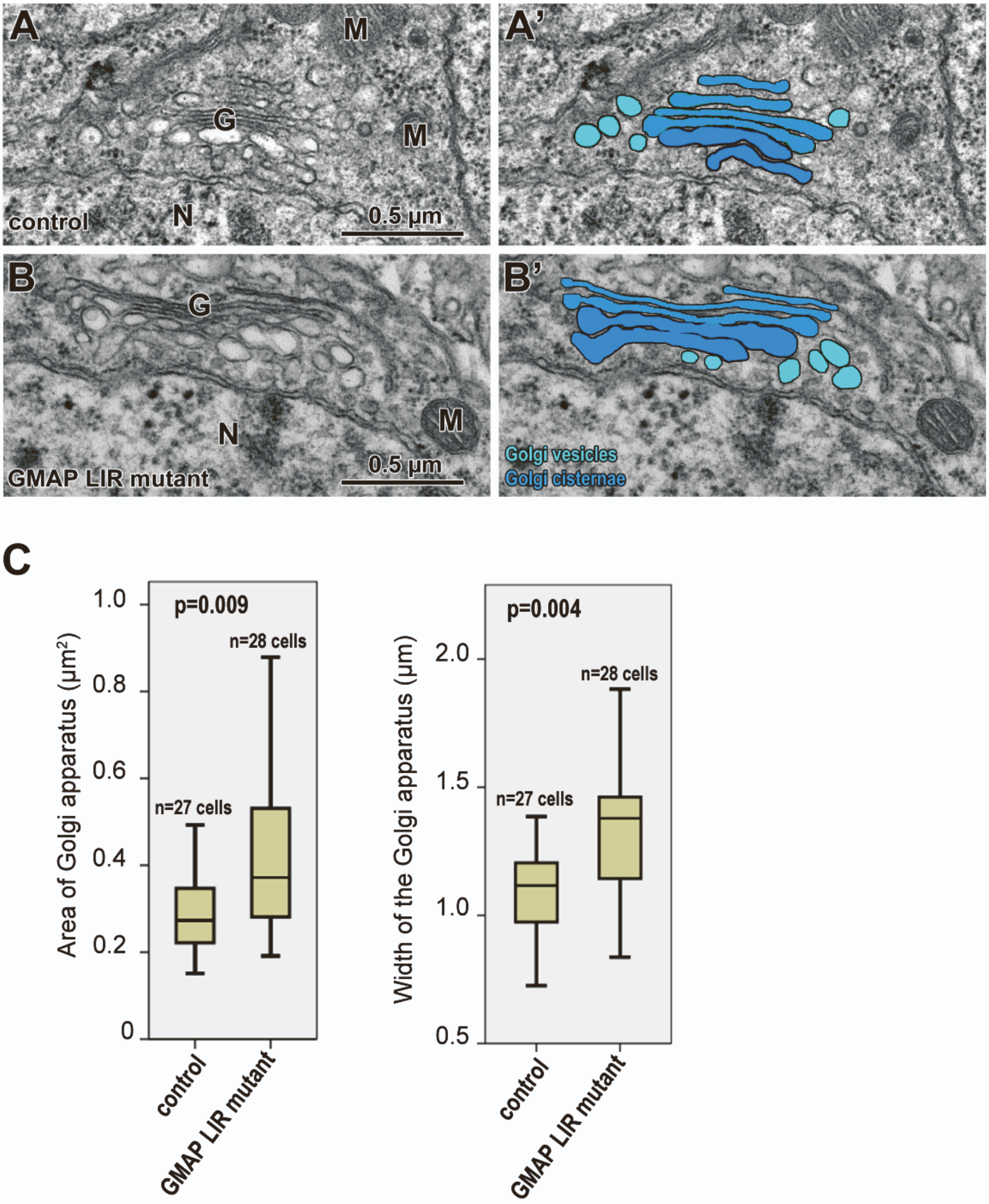

Supplementary Figure 3

Supplementary Figure 3 (Related to Figure 5). GMAP$^{F322A/V325A}$ mutants exhibit elongated Golgi morphology.

Electron micrographs of adult neurons of control **(A)** and GMAP$^{F322A/V325A}$ mutants **(B)**. Enlarged Golgi cisternae are readily apparent in mutants compared to control **(A-C)**. Abbreviations: Golgi (G), Nucleus (N), Mitochondria (M). (N=27 for control and N=28 for mutants). Scale bars: 0.5 µm.

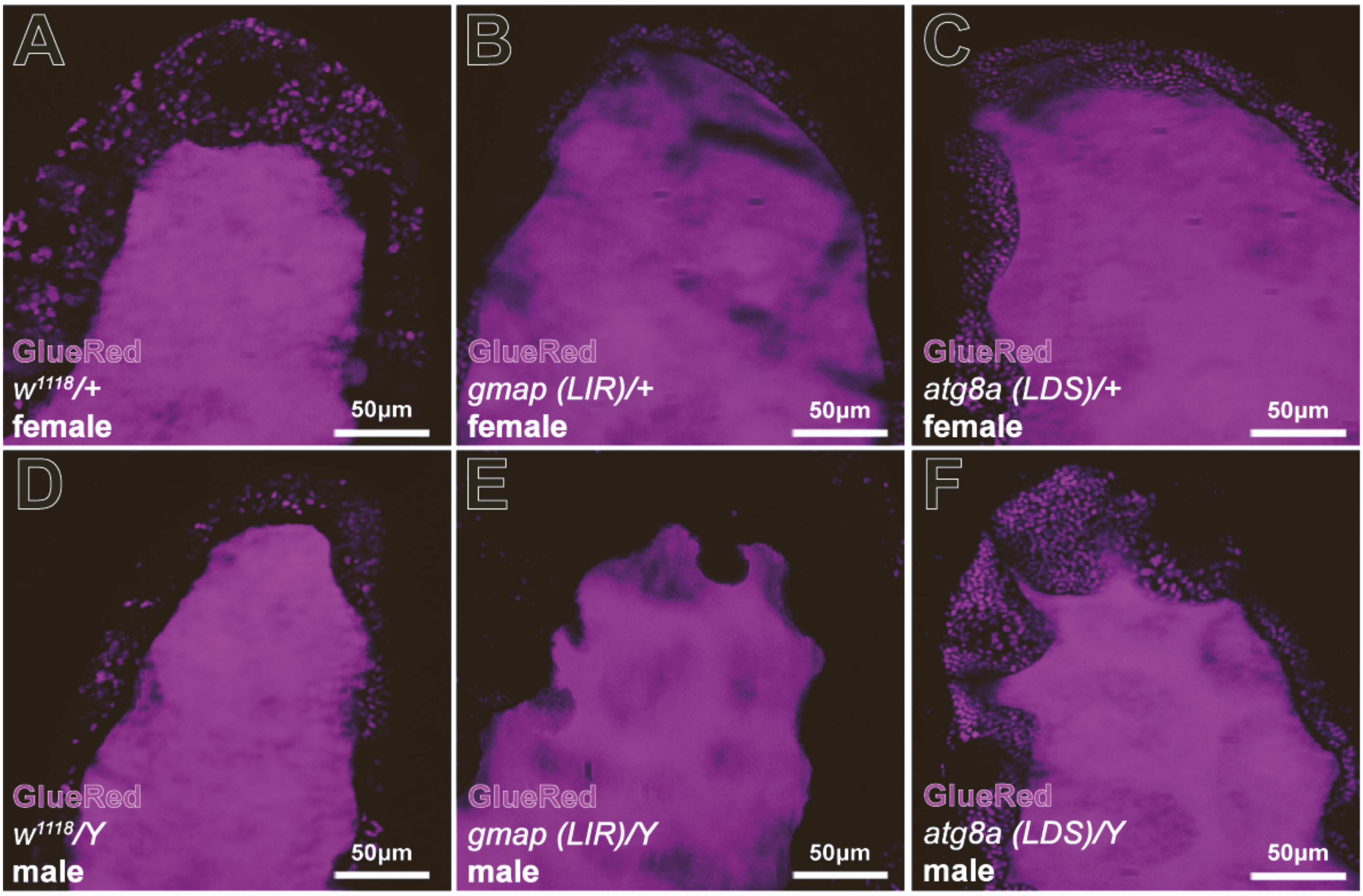

Supplementary Figure 4

Supplementary Figure 4 (Related to Figure 5). GMAP$^{F322A/V325A}$ mutants do not exhibit secretion defects.

The salivary gland-specific (sgs3 promoter-driven) sgs3-DsRed reporter was crossed into the genotypes shown in the images. White prepupal salivary glands are visible in all image panels, and the secreted glue clearly fills the lumen in all cases, with residual granules remaining in cells (but its amount is very variable even in controls). Thus, there is no indication of a secretory defect in GMAP$^{F322A/V325A}$ mutants. Number of biological repeats (N)=3 for all figures.

Genotypes: A: w1118: w^{1118}/+ ; P{Sgs3-DsRed}/+ ; +/+

B: GMAP LIR: GMAP$^{F322A/V325A}$/+; P{Sgs3-DsRed}/+ ; +/+

C: Atg8a LDS: Atg8a$^{K48A/Y49A}$/+; P{Sgs3-DsRed}/+ ; +/+

D: w1118: w^{1118}/Y ; P{Sgs3-DsRed}/+ ; +/+

E: GMAP LIR: GMAP$^{F322A/V325A}$/Y; P{Sgs3-DsRed}/+ ; +/+

F: Atg8a LDS: Atg8a$^{K48A/Y49A}$/Y; P{Sgs3-DsRed}/+ ; +/+

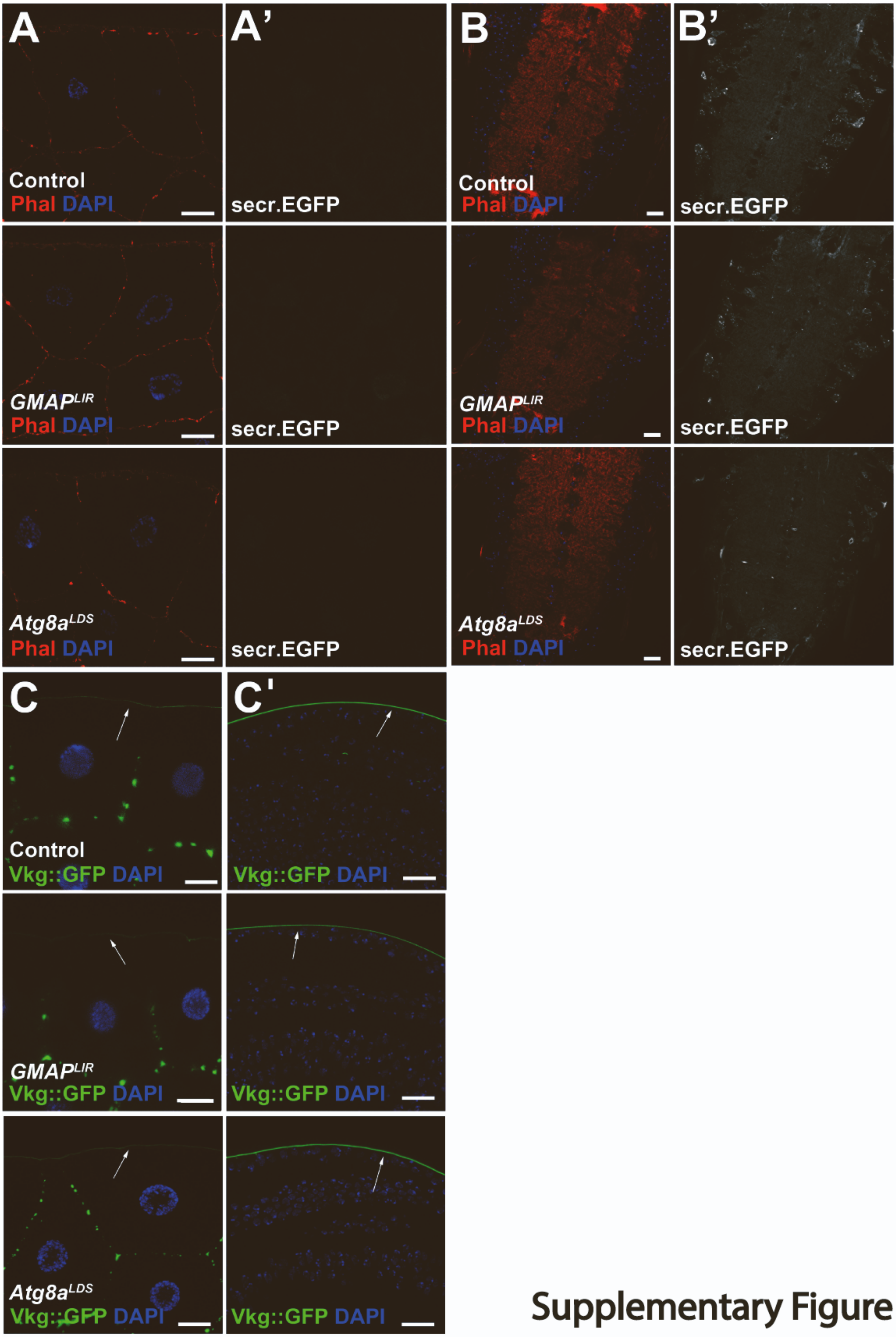

Supplementary Figure 5

Supplementary Figure 5 (Related to Figure 5). GMAP$^{F322A/V325A}$ mutants do not exhibit secretion defects.

(A-B) Fluorescent images show that GMAP $^{F322A/V325A}$ and Atg8a$^{K48A/Y49A}$ mutations do not cause retention of the ubiquitously expressed secr.EGFP secretion reporter in the larval adipose tissue (A) and the larval ventral nerve cord (B). Tissues were counterstained with Phalloidin. **(C)** GMAP $^{F322A/V325A}$ or Atg8a$^{K48A/Y49A}$ did not perturb Collagen IV secretion or caused intracellular retention in the larval adipose tissue (C), or in the wing imaginal disc (C'). Collagen IV is distinguished by the localization of the Vkg::GFP fusion protein. Arrows indicate the basement membranes. Scale bars: 20 μm. Number of biological repeats (N)=3 for all figures. Genotypes: A-B) w^{1118}/Y; UAS-secr.EGFP/+; tub-GAL4/+, GMAP $^{F322A/V325A}$/Y; UAS-secr.EGFP/+; tub-GAL4/+, Atg8a $^{K48A/Y49A}$/Y; UAS-secr.EGFP/+; tub-GAL4/+. C) w^{1118}/Y;P{PTT-un}vkg^{G00454}/+,GMAP $^{F322A/V325A}$/Y;P{PTT-un}vkg^{G00454}/+,
Atg8a $^{K48A/Y49A}$/Y; P{PTT-un}vkg^{G00454}/+.

Supplementary Table S2. The expression level of 29 proteins in mutant Drosophila was higher than that in wild type Drosophila. The proteins are listed by accession number in uniprot, number of protein and peptides, and LFQ (label-free quantification) intensity. These 29 proteins were screened using the following contitions: ratio > 2 and p value < 0.5.

Fasta headers	Number of proteins	Peptides	Razor + unique peptides	Unique peptides	LFQ intensity Atg8a 1	LFQ intensity Atg8a 2	LFQ intensity Atg8a 3	LFQ intensity Atg8a 4	LFQ intensity ATG8a$^{K48A/Y49A}$ 1	LFQ intensity ATG8a$^{K48A/Y49A}$ 2	LFQ intensity ATG8a$^{K48A/Y49A}$ 3	LFQ intensity ATG8a$^{K48A/Y49A}$ 4	LFQ intensity WT1	LFQ intensity WT2	LFQ intensity WT3	LFQ intensity WT4	Average LFQ intensity of Atg8a	Average LFQ intensity of ATG8a$^{K48A/Y49A}$	Average LFQ intensity of WT	Ratio Atg8a/WT	Ratio ATG8a$^{K48A/Y49A}$/WT	p value Atg8a/WT	p value ATG8a$^{K48A/Y49A}$/WT	preferred name	LIR motif	P Anchor
tr|Q7K3E2|Q7K3E2_DROME CG5080, isoform A OS=Drosophila melanogaster GN=CT16297 PE=1 SV=1	1	22	22	22	4.2E+07	9.4E+07	7E+07	5.9E+07	67636000	58255000	29034000	44595000	1.7E+07	2.2E+07	3.6E+07	2.4E+07	66049000	49880000	24680750	2.6761342	2.021008276	0.01159296	0.03557181	CG5080	YES	NO
tr|A0A0B4K6W2|A0A0B4K6W2_DROME Fat facets, isoform E OS=Drosophila melanogaster GN=faf PE=3 SV=	3	18	18	18	8577700	9016300	1.1E+07	1.1E+07	0	8668200	7350900	11604000	0	0	0	0	10005750	6905775	0	#DIV/0!	#DIV/0!	7.5782E-06	0.031221334	faf	YES	NO
tr|Q6IHY5|Q6IHY5_DROME HDC00331 OS=Drosophila melanogaster GN=Dmel\CG34172 PE=1 SV=1	1	2	2	2	1.1E+08	7E+07	7.1E+07	6.4E+07	103160000	87701000	69298000	0	0	0	0	0	78076250	65039750	0	#DIV/0!	#DIV/0!	0.00020042	0.028880452	CG34172	NO	NO
tr|Q8IR72|Q8IR72_DROME FI19011p1 OS=Drosophila melanogaster GN=CG32638-RB PE=1 SV=2	1	3	3	3	1.2E+08	0	9E+07	7.8E+07	61743000	68465000	60786000	0	0	0	0	0	71748250	47748500	0	#DIV/0!	#DIV/0!	0.03018376	0.024545484	CG32638	NO	NO
tr|Q9VDU7|Q9VDU7_DROME LD05707p OS=Drosophila melanogaster GN=Naam PE=2 SV=3	1	7	7	7	1.4E+07	1.5E+07	1.5E+07	1.2E+07	11364000	13802000	13021000	12240000	1.3E+07	0	0	0	13968250	12606750	3127500	4.466267	4.030935252	0.01460035	0.024337065	Naam	NO	NO
tr|Q9VLV9|Q9VLV9_DROME Proctolin OS=Drosophila melanogaster GN=Proc PE=2 SV=2	1	2	2	2	8703600	1.2E+07	8385500	8736000	6878200	4526400	7787400	4594800	3536500	3237600	3024800	0	9499525	5946700	2449725	3.8777924	2.427496964	0.00115312	0.023807031	Proc	NO	NO
tr|A0A0B4KEK7|A0A0B4KEK7_DROME PI31, isoform D OS=Drosophila melanogaster GN=PI31 PE=4 SV=1;s	2	4	4	4	1.1E+07	1.8E+07	1.8E+07	1.2E+07	14105000	17153000	16649000	14322000	1E+07	0	1E+07	8975700	15120500	15557250	7441175	2.0320044	2.090698042	0.04979791	0.021273536	PI31	YES	NO
tr|Q9VIX4|Q9VIX4_DROME Acyl-coenzyme A oxidase OS=Drosophila melanogaster GN=Dmel\CG17544 PE=1	1	20	20	20	4.9E+07	4.3E+07	7.8E+07	8.9E+07	24183000	27366000	17354000	49323000	5441200	8901300	5936100	8904100	64562500	29556500	7295675	8.8494211	4.051235835	0.00210498	0.018795974	CG17544	YES	NO
tr|Q9VZF1|Q9VZF1_DROME CG1309 OS=Drosophila melanogaster GN=Dmel\CG1309 PE=1 SV=1	1	9	9	9	1.6E+07	1.8E+07	1.6E+07	2.4E+07	15907000	23462000	16287000	13676000	9482300	1E+07	0	0	18514250	17333000	4914325	3.7674045	3.527035758	0.0077624	0.012796017	CG1309	NO	NO
sp|P02515|HSP22_DROME Heat shock protein 22 OS=Drosophila melanogaster GN=Hsp22 PE=1 SV=4	1	9	9	9	1.1E+08	1.4E+08	5.8E+07	9.8E+07	17198000	14414000	19554000	9968500	0	7825500	0	6437400	102654000	15283625	3565725	28.789096	4.286260157	0.00137242	0.00707916	Hsp22	YES	NO
tr|A0A0B4LH23|A0A0B4LH23_DROME RIC-3, isoform V OS=Drosophila melanogaster GN=RIC-3 PE=4 SV=1	15	8	8	8	1.8E+07	1.7E+07	1.8E+07	1.4E+07	24466000	16090000	22908000	21190000	0	1.5E+07	0	0	16832750	21163500	3699000	4.5506218	5.721411192	0.01354687	0.005457873	RIC-3	NO	NO
tr|Q9VGE7|Q9VGE7_DROME Beta-galactosidase OS=Drosophila melanogaster GN=Ect3 PE=1 SV=1	1	9	9	9	2.2E+07	1.6E+07	1.7E+07	1.5E+07	25924000	27301000	20987000	16756000	1.2E+07	7952400	9202500	0	17569000	22742000	7319475	2.4003088	3.107053443	0.01395916	0.004790721	Ect3	NO	NO
tr|Q7K3B7|Q7K3B7_DROME LD40177p OS=Drosophila melanogaster GN=CG11208 PE=1 SV=1	1	14	14	14	3.8E+07	1.8E+07	3.5E+07	3.7E+07	21592000	20363000	24913000	28255000	1.2E+07	9489700	0	0	31894750	23780750	5412925	5.8923318	4.393327083	0.00300544	0.002326007	CG11208	YES	NO
tr|Q7K5M6|Q7K5M6_DROME GH04176p OS=Drosophila melanogaster GN=Sip1 PE=1 SV=1	1	9	9	9	1.7E+07	1.2E+07	1.4E+07	1.2E+07	10023000	12230000	15515000	12410000	0	0	0	6741300	13654500	12544500	1685325	8.1019981	7.443371457	0.00147858	0.001739584	Sip1	NO	YES
sp|Q9VG97|GSTD3_DROME Inactive glutathione S-transferase D3 OS=Drosophila melanogaster GN=GstD3 PE=	1	7	7	7	1.8E+07	1.8E+07	3.1E+07	2.7E+07	14837000	19676000	27681000	20322000	0	6126200	0	0	23691500	20629000	1531550	15.468969	13.4693611	0.00094562	0.000784903	GstD3	YES	NO
tr|Q53XG2|Q53XG2_DROME Division abnormally delayed, isoform B OS=Drosophila melanogaster GN=dally PE	2	7	7	7	6447800	5766300	7550500	5899000	6542700	8155700	5551100	3985100	0	0	0	0	6415900	6058650	0	#DIV/0!	#DIV/0!	4.0691E-06	0.000449452	dally	YES	YES
tr|Q9VBU6|Q9VBU6_DROME RE24638p OS=Drosophila melanogaster GN=CG11857 PE=1 SV=1	1	6	6	1	6.2E+07	7.6E+07	7.1E+07	6.2E+07	53529000	56704000	65897000	51294000	3.1E+07	3.2E+07	2.4E+07	2.5E+07	67521500	56856000	28036750	2.4083212	2.027909797	6.9108E-05	0.000258626	CG11857	YES	NO
sp|P14199|REF2P_DROME Protein ref(2)P OS=Drosophila melanogaster GN=ref(2)P PE=1 SV=2	1	17	17	17	4.6E+08	5.6E+08	3.9E+08	3.5E+08	87102000	68651000	87945000	51836000	7243400	3432200	4237200	2699400	439115000	73883500	4403050	99.729733	16.7800729	9.4014E-05	0.000198945	ref(2)P	YES	YES
sp|Q9VIU7|DPM1_DROME Probable dolichol-phosphate mannosyltransferase OS=Drosophila melanogaster GN=C	1	5	5	5	1.6E+07	1.5E+07	1.6E+07	1.7E+07	16092000	13953000	17980000	9821500	0	0	0	0	16126750	14461625	0	#DIV/0!	#DIV/0!	1.2987E-08	0.000170861	CG10166	YES	NO
tr|Q9W3Q1|Q9W3Q1_DROME GM14286p OS=Drosophila melanogaster GN=Pdp PE=1 SV=1	1	9	9	9	0	1.3E+07	6948500	9111500	6784100	8983800	8088400	5764200	0	0	0	0	7285250	7405125	0	#DIV/0!	#DIV/0!	0.03759869	4.52877E-05	Pdp	YES	NO
tr|Q9VXU2|Q9VXU2_DROME Golgi microtubule-associated protein, isoform A OS=Drosophila melanogaster GN=	2	21	21	21	2.8E+07	2.4E+07	1.8E+07	1.5E+07	13660000	11006000	15479000	10335000	0	0	0	0	21619000	12620000	0	#DIV/0!	#DIV/0!	0.00030631	4.20151E-05	Gmap	YES	YES
tr|M9PCN6|M9PCN6_DROME Numb, isoform D OS=Drosophila melanogaster GN=numb PE=4 SV=1;sp|P16554	2	6	6	6	8211600	1.2E+07	9367700	7753400	6065800	8246400	8399600	6404500	0	0	0	0	9419675	7279075	0	#DIV/0!	#DIV/0!	9.7701E-05	2.04783E-05	numb	NO	NO
tr|Q9VG92|Q9VG92_DROME Glutathione S transferase D8 OS=Drosophila melanogaster GN=GstD8 PE=2 SV=1	1	6	6	6	8031500	8481800	7199800	6258700	7957600	9058900	11393000	8589400	0	0	0	0	7492950	9249725	0	#DIV/0!	#DIV/0!	4.9191E-06	1.72223E-05	GstD8	YES	NO
tr|Q9VN39|Q9VN39_DROME CG9775, isoform A OS=Drosophila melanogaster GN=CG9775 PE=1 SV=1	2	5	5	5	5332800	7418600	0	4784100	6151200	6067800	7774500	5663000	0	0	0	0	4383875	6414125	0	#DIV/0!	#DIV/0!	0.03130612	9.1257E-06	CG9775	NO	YES
tr|A1ZB69|A1ZB69_DROME Glutathione S transferase E4 OS=Drosophila melanogaster GN=GstE4 PE=3 SV=1	1	9	9	9	1.1E+08	1.2E+08	8E+07	9.4E+07	149890000	152980000	141210000	123420000	4.5E+07	4E+07	4.8E+07	4.8E+07	100038250	141875000	45458750	2.2006379	3.120961311	0.00085178	8.33344E-06	GstE4	YES	NO
tr|A4V488|A4V488_DROME Inosine-5-monophosphate dehydrogenase OS=Drosophila melanogaster GN=ras PE=	2	7	7	7	1.2E+07	0	7820000	7853100	11928000	9137300	9035200	9516500	0	0	0	0	6900275	9904250	0	#DIV/0!	#DIV/0!	0.03256616	6.71249E-06	ras	YES	YES
tr|A0A0B4JD21|A0A0B4JD21_DROME Alkylglycerone-phosphate synthase OS=Drosophila melanogaster GN=A	2	8	8	8	1.9E+07	1.6E+07	1.8E+07	1.8E+07	10879000	12045000	13933000	14633000	0	0	0	0	17562750	12872500	0	#DIV/0!	#DIV/0!	1.5408E-07	5.61398E-06	CG10253	NO	NO
tr|Q86BQ3|Q86BQ3_DROME Uncharacterized protein, isoform A OS=Drosophila melanogaster GN=Dmel\CG132	3	8	8	7	1.1E+07	8370200	9259500	7679400	9506100	8262800	10817000	8498500	0	0	0	0	9120525	9271100	0	#DIV/0!	#DIV/0!	1.9801E-05	3.86807E-06	CG13284	YES	NO
tr|A0A0B4LGN1|A0A0B4LGN1_DROME Inositol 1,4,5,-tris-phosphate receptor, isoform C OS=Drosophila melan	2	18	18	18	7885300	8429100	8443700	8662200	7844000	7670100	7321500	8500400	0	0	0	0	8355075	7834000	0	#DIV/0!	#DIV/0!	4.04E-09	6.57104E-08	Itp-r83A	YES	NO

Supplementary Table 2 (Related to Figure 2). List of proteins that significantly accumulate in Atg8a ^{KG07569} and Atg8a$^{K48A/Y49A}$ mutants.

Results showing the expression level of 29 proteins that are upregulated in Atg8a ^{KG07569} and Atg8a$^{K48A/Y49A}$ mutants. The proteins are listed by accession number in Uniprot, number of protein and peptides, and LFQ (label-free quantification) intensity. These 29 proteins were screened using the following conditions: ratio > 2 and p value < 0.5.
