# Supplementary material for: GMAP is an Atg8a-interacting protein that regulates Golgi turnover in Drosophila
Source: Cell Rep. 2022 May 31;39(9):110903. doi: 10.1016/j.celrep.2022.110903 (PMC9637997; doi:10.1016/j.celrep.2022.110903)
Supplement: Document S2. Article plus supplemental information [file mmc3.pdf]

Cell Reports

Article

GMAP is an Atg8a-interacting protein that regulates Golgi turnover in *Drosophila*

Graphical abstract

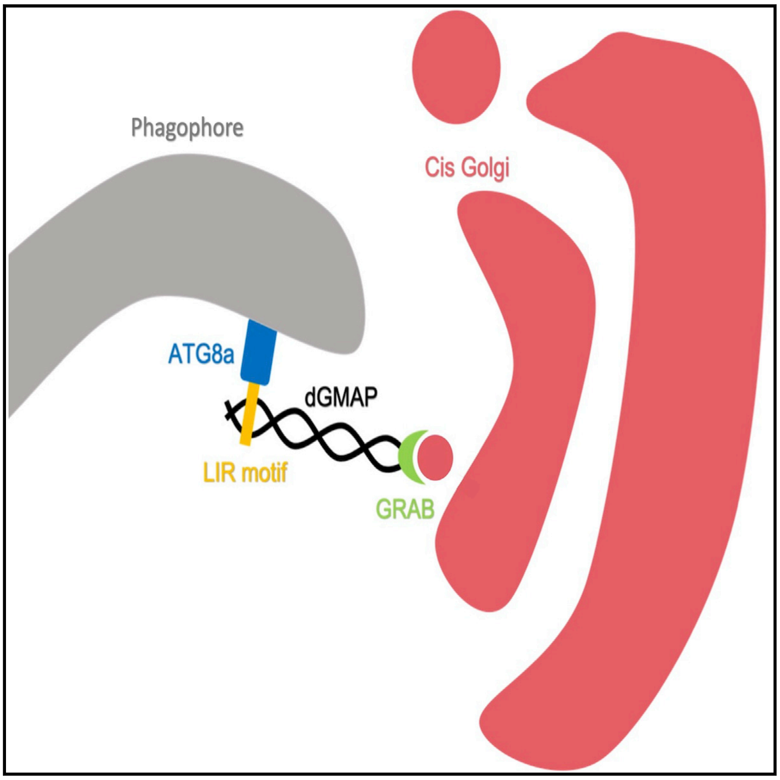

Authors

Ashrafur Rahman, Peter Lőrincz, Raksha Gohel, ..., Yan Zhang, Gábor Juhász, Ioannis P. Nezis

Correspondence

i.nezis@warwick.ac.uk

In brief

Rahman et al. create Atg8a-LDS (LIR motif docking site) mutants in *Drosophila*. They show that Atg8a-LDS mutants accumulate autophagy substrates and have reduced lifespan. Using quantitative proteomics, they identify Golgi protein GMAP as a LIR motif-containing protein that interacts with Atg8a. They show that GMAP LIR mutants exhibit elongated Golgi morphology.

Highlights

- Atg8a-LDS mutants accumulate autophagy substrates and have reduced lifespan
- Quantitative proteomics identifies accumulation of GMAP in Atg8a-LDS mutants
- GMAP interacts with Atg8a via a LIR motif
- Atg8a-LDS and GMAP LIR motif mutants exhibit elongated Golgi morphology

Rahman et al., 2022, Cell Reports *39*, 110903
May 31, 2022 © 2022 The Author(s).
https://doi.org/10.1016/j.celrep.2022.110903

CellPress

Cell Reports

CellPress
OPEN ACCESS

Article

GMAP is an Atg8a-interacting protein that regulates Golgi turnover in *Drosophila*

Ashrafur Rahman,[1] Peter Lőrincz,[2] Raksha Gohel,[1] Anikó Nagy,[2] Gábor Csordás,[3] Yan Zhang,[1,4] Gábor Juhász,[2,3] and Ioannis P. Nezis[1,5,*]

[1]School of Life Sciences, University of Warwick, CV4 7AL Coventry, UK
[2]Department of Anatomy, Cell and Developmental Biology, Eötvös Loránd University, Budapest, Hungary
[3]Institute of Genetics, Biological Research Centre, Szeged, Hungary
[4]State Key Laboratory of Silkworm Genome Biology, Biological Science Research Center, Southwest University, Chongqing 400715, China
[5]Lead contact
*Correspondence: i.nezis@warwick.ac.uk
https://doi.org/10.1016/j.celrep.2022.110903

SUMMARY

Selective autophagy receptors and adapters contain short linear motifs called LIR motifs (LC3-interacting region), which are required for the interaction with the Atg8-family proteins. LIR motifs bind to the hydrophobic pockets of the LIR motif docking site (LDS) of the respective Atg8-family proteins. The physiological significance of LDS docking sites has not been clarified *in vivo*. Here, we show that Atg8a-LDS mutant *Drosophila* flies accumulate autophagy substrates and have reduced lifespan. Using quantitative proteomics to identify the proteins that accumulate in Atg8a-LDS mutants, we identify the *cis*-Golgi protein GMAP (Golgi microtubule-associated protein) as a LIR motif-containing protein that interacts with Atg8a. GMAP LIR mutant flies exhibit accumulation of Golgi markers and elongated Golgi morphology. Our data suggest that GMAP mediates the turnover of Golgi by selective autophagy to regulate its morphology and size via its LIR motif-mediated interaction with Atg8a.

INTRODUCTION

Autophagy is an evolutionarily conserved process where cells degrade their own cellular material. It is involved in protein and organelle degradation and plays an essential role in both cellular and whole-animal homeostasis. Autophagy is a cellular response in nutrient starvation but also responsible for removing aggregated proteins, damaged organelles, and invading bacteria and viruses (Lamb et al., 2013; Randow and Youle, 2014). There are various types of autophagy, such as macroautophagy, microautophagy, and chaperone-mediated autophagy (Lamb et al., 2013). During macroautophagy, there is sequestration of cellular material into double-membrane vesicles called autophagosomes. The autophagosomes are subsequently fused with the lysosomes where the sequestered cargoes are degraded by lysosomal hydrolases. The products of degradation are transported back into the cytoplasm through lysosomal membrane permeases and can be reused by the cell (Lamb et al., 2013). Although it was initially believed that autophagy occurs randomly inside the cell, it is now established that sequestration and degradation of cytoplasmic material by autophagy can be selective through receptor and adapter proteins (Randow and Youle, 2014; Johansen and Lamark, 2020).

The core autophagic machinery, including Atg8 proteins, is highly conserved from yeast to humans (Johansen and Lamark, 2020). In yeast, there is a single Atg8 protein involved in selective autophagy. The evolution of multicellular organisms gave rise to two families of Atg8 proteins; MAP1LC3, often called LC3, and GABARAP. In humans the Atg8 family of proteins consists of seven proteins: LC3A, LC3B, LC3B2, LC3C, GABARAP, GABARAPL1, and GABARAPL2 (Johansen and Lamark, 2020). Atg8 proteins were discovered to be a central player in selective autophagy through their interaction with LIR motif-containing proteins. The LIR motif was initially characterized in p62 (mammals) and ATG19 (yeast) (Johansen and Lamark, 2020). Selective autophagy receptors and adapters contain short linear motifs LIR motifs (LC3-interacting region motifs), LC3 recognition sequences (LRS), or Atg8-interacting motifs (AIM), which are required for the interaction with Atg8-family proteins (Atg8/LC3/GABARAP) (Pankiv et al., 2007; Ichimura et al., 2008; Noda et al., 2010). LIR motif-containing proteins (LIRCPs) bind via their LIR motif to the hydrophobic pocket 1 (HP1) and hydrophobic pocket 2 (HP2) of the LIR docking site (LDS) of the respective Atg8 protein (Johansen and Lamark, 2020). Different variations of LIR motifs have a preference to different forms of ATG8. For example, some LIR motifs exclusively bind to GABRAP while others preferentially bind to LC3 proteins (Rogov et al., 2017; Wirth et al., 2019). In simpler multicellular organisms, such as *Drosophila* there are only two Atg8 proteins (Atg8a and Atg8b). *Drosophila* Atg8b expression is only observed in the male germline and it is required for male fertility independent of its lipidation and autophagy (Jipa et al., 2021). Sequence analysis of Atg8a is almost identical with Atg8b and suggests that Atg8a and Atg8b have LDS sites (Jipa et al., 2021). Atg8 proteins

© 2022 The Author(s).
This is an open access article under the CC BY license (http://creativecommons.org/licenses/by/4.0/).

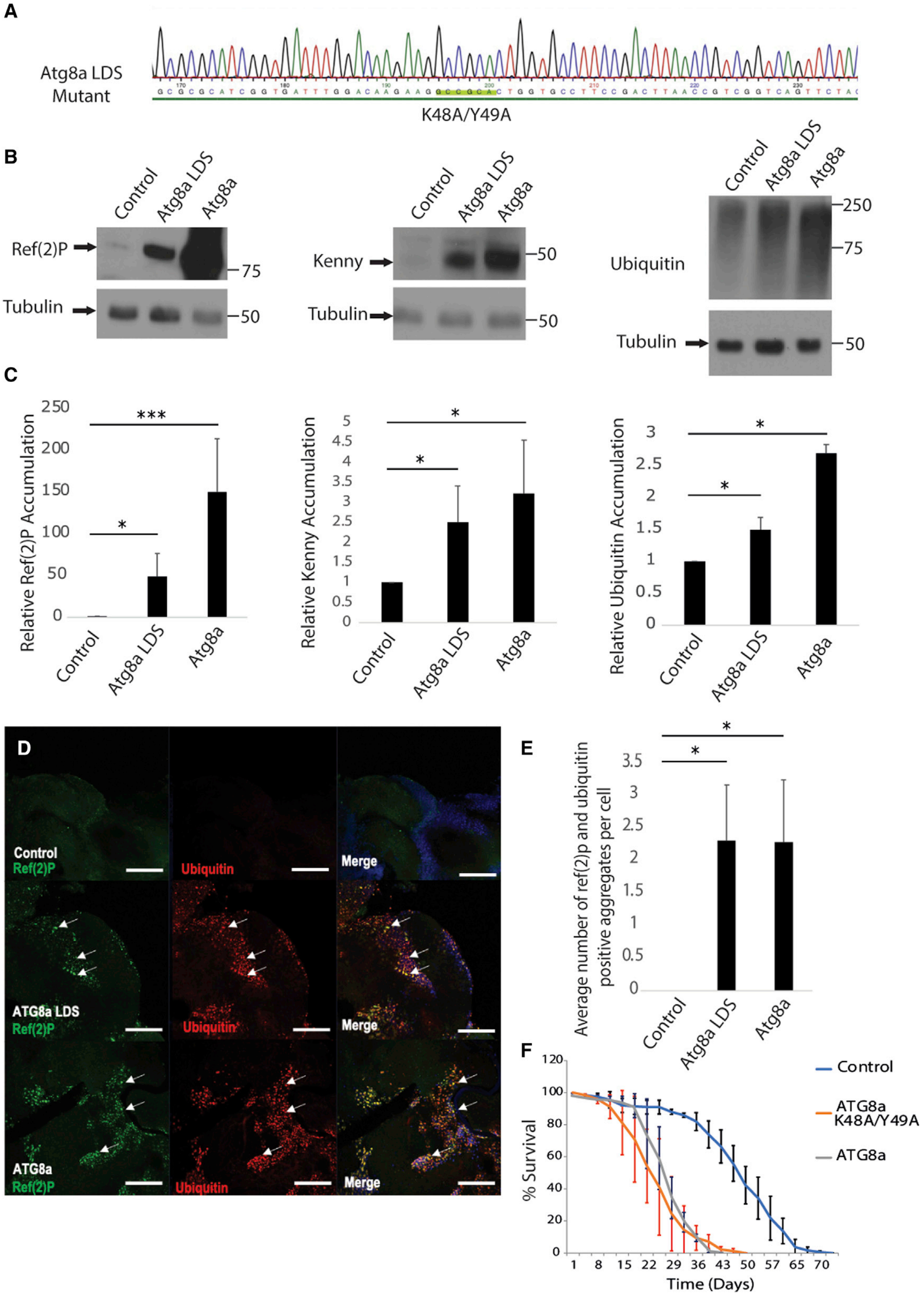

(legend on next page)

also contain a UIM (ubiquitin-interacting motif) docking site (UDS), which mediates another type of interaction that is LIR motif/LDS independent (Marshall et al., 2019). Despite the growing identification of selective autophagy receptors and adapters in mammals, the regulation and mechanisms of action of selective autophagy receptors and adapters, and the physiological significance of Atg8's LDS docking site, are poorly described in the fruit fly *Drosophila melanogaster*.

Selective autophagy mediates the degradation of organelles (Anding and Baehrecke, 2017). However, autophagic degradation of the Golgi apparatus is not well studied (Mijaljica et al., 2006; Lu et al., 2020; De Tito et al., 2020; Nthiga et al., 2021). In this study, we created Atg8a LDS mutant flies using CRISPR. Atg8a LDS mutants exhibit a similar phenotype with Atg8a protein null mutant flies, including accumulation of ubiquitin-positive aggregates and reduced lifespan. To identify the proteins that accumulate in these mutants we performed quantitative proteomics. We identified GMAP (Golgi microtubule-associated protein), a *cis*-Golgi protein, as an Atg8a-interacting protein that regulates Golgi turnover.

RESULTS

Generation and characterization of Atg8a$^{K48A/Y49A}$ mutants

To elucidate the physiological significance of Atg8a-LIRCPs interactions in *Drosophila*, we used CRISPR to generate Atg8a$^{K48A/Y49A}$ (Atg8a LDS) mutants (Figure S1A). Atg8a$^{K48A/Y49A}$ flies have two point mutations (K48A and Y49A) within the hydrophobic LDS of Atg8a that abolish interactions with LIRCPs (Johansen and Lamark, 2020). The K48A/Y49A mutation was confirmed using genomic sequencing (Figure 1A). Expression of the Atg8a protein is observed in the wild-type flies as well as the Atg8a$^{K48A/Y49A}$ mutants (Figure S1B). This shows that Atg8a is successfully being expressed in Atg8a$^{K48A/Y49A}$ mutant flies. Atg8a^{KG07569} mutant flies were used as a negative control as they do not express Atg8a protein (protein null mutants) (Nezis et al., 2008) (Figure S1B). Atg8a$^{K48A/Y49A}$ flies are viable. To examine whether Atg8a$^{K48A/Y49A}$ flies accumulate LIRCPs, we performed western blotting for Ref(2)P and Kenny, two proteins that have been shown to interact with Atg8a via LIR motifs (Jain et al., 2015; Nezis et al., 2008; Tusco et al., 2017). Western blotting analysis showed that Ref(2)P and Kenny, as well as ubiquitinated proteins, accumulated in Atg8a$^{K48A/Y49A}$ mutant flies (Figures 1B, 1C, and S1C), indicating that the LIR motif/LDS interaction is important for their degradation by autophagy. Expression of 3xmCherry-Atg8a in Atg8a$^{K48A/Y49A}$ mutant background was sufficient to rescue accumulation of Ref(2)P (Figure S1D). We further used immunofluorescence confocal microscopy to determine the expression of Ref(2)P in adult *Drosophila* brain and found that Atg8a$^{K48A/Y49A}$ mutant flies showed a significant increase in the number of Ref(2)P- and ubiquitin-positive puncta (Figures 1D and 1E). In addition, we observed that Atg8a$^{K48A/Y49A}$ mutation did not negatively affect bulk autophagy (Figures S1E and S1F). We also observed that Atg8a$^{K48A/Y49A}$ mutant flies have a short lifespan, which was similar to that of Atg8a^{KG07569} mutant flies (Figure 1F). All together, these data show that Atg8a's LDS docking site is physiologically important for the function of Atg8a protein to selectively degrade LIRCPs.

Quantitative proteomics analysis of Atg8a$^{K48A/Y49A}$ mutants

To identify the proteins that accumulate in Atg8a$^{K48A/Y49A}$ mutants, we collected 2-week-old fly heads and performed quantitative proteomics analysis. Analysis by LC-MS/MS identified 3,036, 2,342, and 2,468 proteins from wild-type, Atg8a^{KG07569}, and Atg8a$^{K48A/Y49A}$ mutant fly heads, respectively (Table S1). Principal-component analysis divided the 12 protein samples into 3 obvious groups: wild-type, Atg8a^{KG07569}, and Atg8a$^{K48A/Y49A}$ mutant (Figure 2A). To identify the upregulated proteins in mutant flies, we set the cut-off p value as <0.05, together with a difference of more than 2-fold between mutant and wild-type flies (Table S2). Twenty-nine proteins passed these two criteria and showed upregulated expression in both Atg8a^{KG07569} and Atg8a$^{K48A/Y49A}$ mutants (Figures 2B; Table S2). Among them, Ref(2)P (Figure 2C) has been already shown to have a functional LIR motif (Jain et al., 2015). GMAP was also shown to be significantly upregulated in Atg8a$^{K48A/Y49A}$ flies (Figure 2C). We therefore focused to further characterize the function of GMAP in selective autophagy.

GMAP is an Atg8a-interacting protein

GMAP is a *cis*-Golgi protein that has a role in anterograde transport and Golgi organization *in vivo* (Friggi-Grelin et al., 2006; Sinka et al., 2008). To verify the proteomics data, we tested if GMAP accumulates in Atg8a LDS mutants. Western blot analysis showed that GMAP is accumulated in Atg8a^{KG07569} and Atg8a$^{K48A/Y49A}$ mutant flies compared with wild-type flies (Figures 3A and 3B). We further used immunofluorescence confocal microscopy to determine the expression pattern of GMAP in adult *Drosophila* brain. We observed that there is a significant increase in the number of GMAP- and

Figure 1. Characterization of Atg8a$^{K48A/Y49A}$ (LDS) mutant flies

(A) Genomic DNA from Atg8a $^{K48A/Y49A}$ mutant flies was extracted, and the sequenced results confirmed the successful incorporation of the K48A/Y49A mutation.

(B) Wild-type, Atg8a^{KG07569}, and Atg8a$^{K48A/Y49A}$ mutant flies were aged for 2 weeks. Western blot analysis of lysates from whole flies showed that Ref(2)P, Kenny, and ubiquitin were accumulated in both Atg8a^{KG07569} and Atg8a$^{K48A/Y49A}$ mutant flies.

(C) Quantification of the western blottings in (B) shows significant accumulations of the aforementioned proteins in both Atg8a^{KG07569} and Atg8a$^{K48A/Y49A}$ mutant flies.

(D and E) Confocal images from 2-week-old adult brains. Ref(2)P (green) and ubiquitin (red) aggregates (arrows) can be seen in Atg8a^{KG07569} and Atg8a$^{K48A/Y49A}$ mutant flies and not in wild-type flies. DNA was dyed with Hoechst (blue). Scale bars, 60 μm.

(F) Survival test of wild-type, Atg8a ^{KG07569}, and Atg8a$^{K48A/Y49A}$ mutant flies. The results show that Atg8a and Atg8a$^{K48A/Y49A}$ mutant flies have a short lifespan. Bar charts show means $\pm$ SD. Statistical significance was determined using two-tailed Student's t test. $^{*}p < 0.05$, $^{**}p < 0.01$, $^{***}p < 0.001$. Number of biological repeats (N = 3 for all figures). Genotypes for all figures: control: w^{1118}/Y, Atg8a LDS: Atg8a $^{K48A/Y49A}$/Y, Atg8a: Atg8a ^{KG07569}/Y.

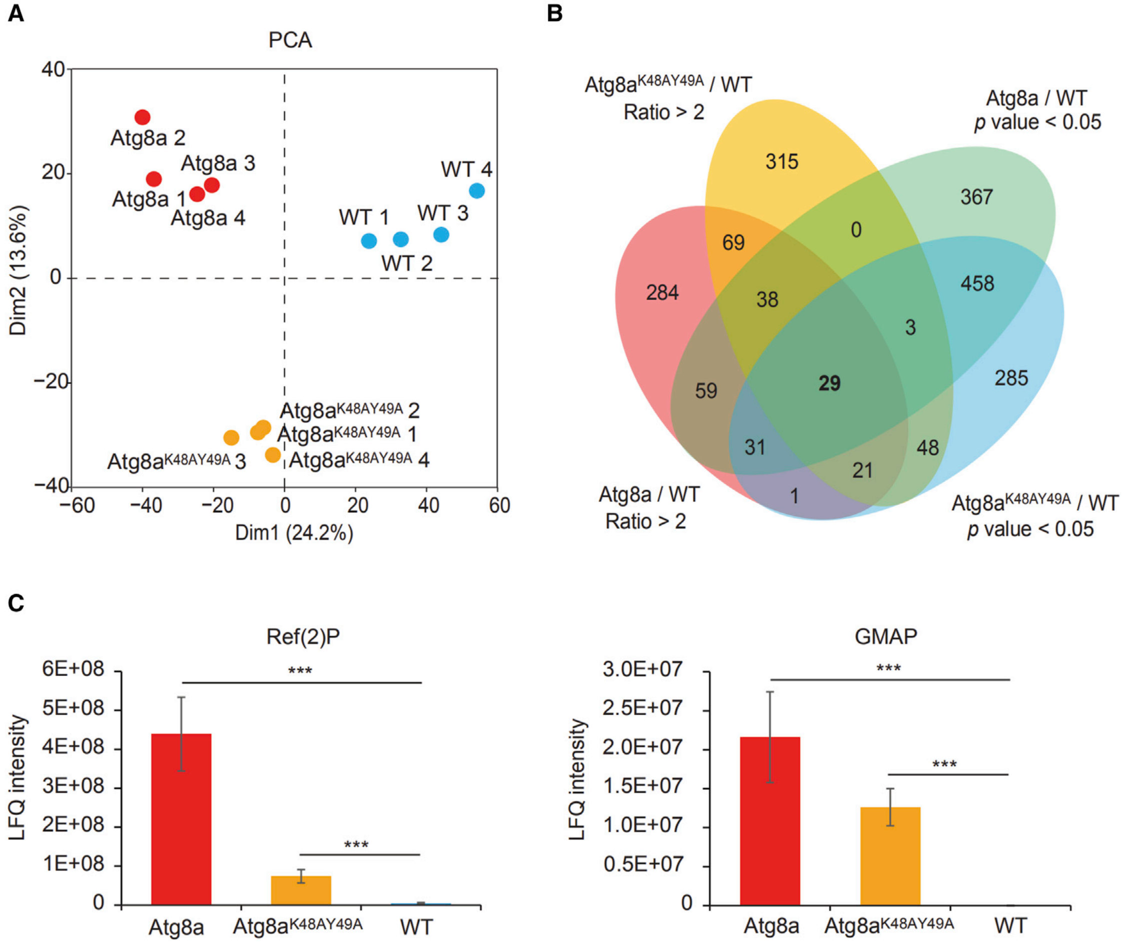

Figure 2. Quantitative proteomic analysis of Atg8a$^{K48A/Y49A}$ mutant flies

(A) Principal-component analysis (PCA) of wild-type, Atg8a^{KG07569}, and Atg8a$^{K48A/Y49A}$ mutant adult *Drosophila* heads. Two-week-old male flies were selected and their heads were collected to perform the proteomic analysis. Four biological replicates were performed for each sample. PCA divided the 12 protein samples into three obvious groups.

(B) Venn diagram representing upregulated proteins in *Drosophila* mutant flies. The cut-off p value was set as <0.05 together with a difference of more than 2-fold between mutant and wild-type *Drosophila* heads. Twenty-nine proteins passed these two criteria and showed upregulated expression in both of Atg8a^{KG07569} and Atg8a$^{K48A/Y49A}$ mutants.

(C) The iBAQ intensity is used to show upregulation of Ref(2)P and GMAP. Bar charts show means ± SD. *p < 0.05, **p < 0.01, ***p < 0.001. Genotypes for all figures: wild-type: w^{1118}/Y, Atg8a LDS: Atg8a $^{K48A/Y49A}$/Y, Atg8a: Atg8a ^{KG07569}/Y.

ubiquitin-positive structures in the adult brain of Atg8a^{KG07569} and Atg8a$^{K48A/Y49A}$ mutant flies compared with wild-type files (Figure 3C). In addition, the size of GMAP puncta was significantly increased in Atg8a^{KG07569} and Atg8a$^{K48A/Y49A}$ mutant flies (Figures 3C and 3D). These data suggest that selective autophagy regulates the size and morphology of the Golgi apparatus.

GMAP is a coiled-coil protein that has 12 coiled-coil domains and a GRAB domain (Friggi-Grelin et al., 2006; Sinka et al., 2008) (Figure 4A). We used iLIR software (Kalvari et al., 2014; Jacomin et al., 2016) to predict functional LIR motifs in the GMAP protein. GMAP has a predicted LIR motif at position 320–325 with the sequence DEFIVV (Figure 4B). To examine whether GMAP interacts with Atg8a and has a functional LIR motif, we performed GST pulldowns and confirmed the direct interaction between GMAP and Atg8a (Figures 4C and 4D). Atg8a-LDS showed significantly decreased binding to GMAP (Figures 4C and 4D). In addition, point mutations of the GMAP LIR motif in positions 322 and 325 by alanine substitutions of the aromatic and hydrophobic residues (F322A and V325A) reduced its binding to Atg8a (Figures 4E and 4F). These results show that GMAP interacts with Atg8a and that LIR motif at position 320–325 is important for this interaction.

GMAP mediates Golgi turnover

Since GMAP is a Golgi protein that interacts with Atg8a we examined if it regulates the autophagic degradation of the Golgi complex. We observed that GMAP colocalizes with Atg8a during starvation (Figure 5A). We also observed that the Golgi marker GM130 accumulates in Atg8a$^{K48A/Y49A}$ and Atg8a^{KG07569} mutants, suggesting that autophagy regulates Golgi turnover

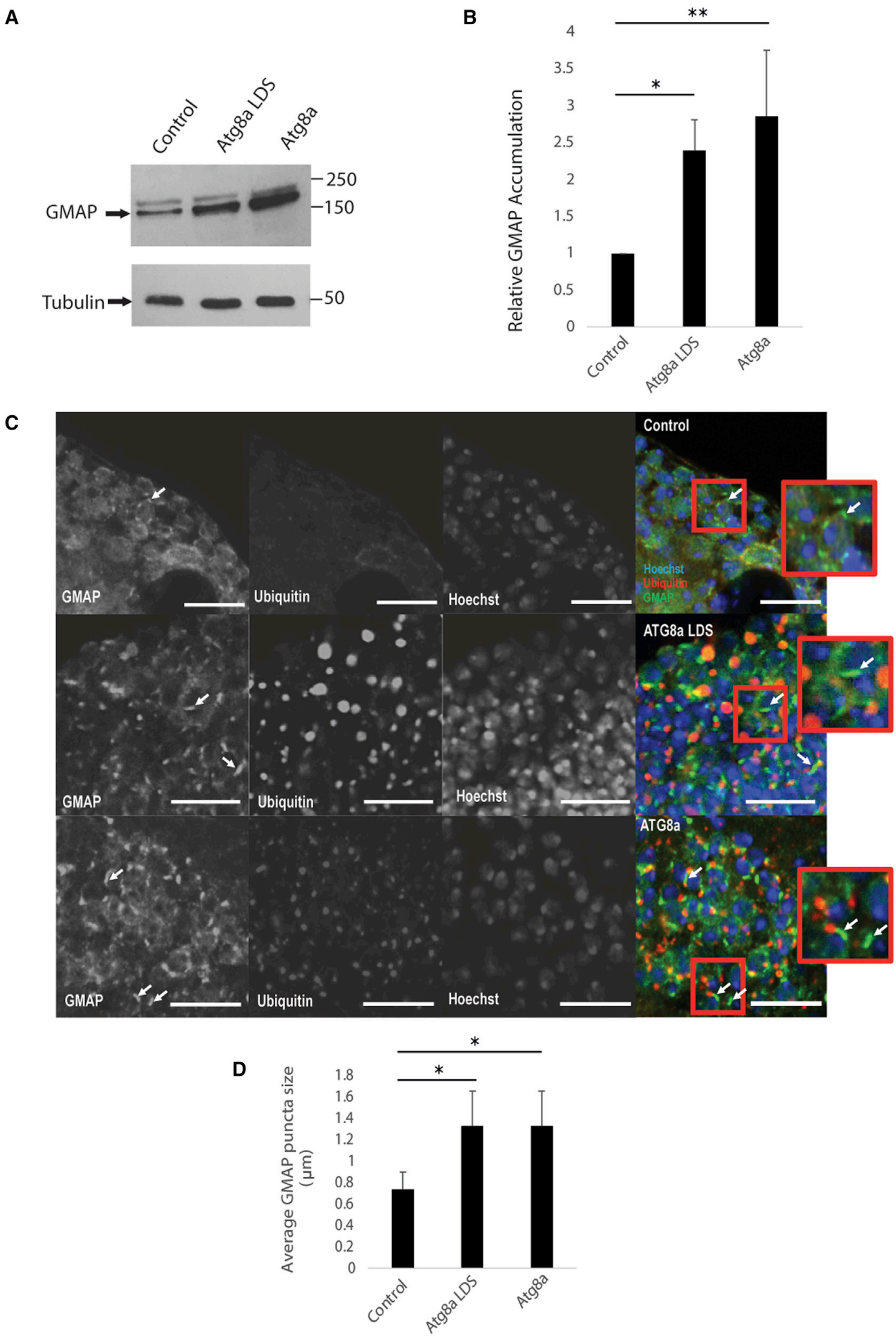

(legend on next page)

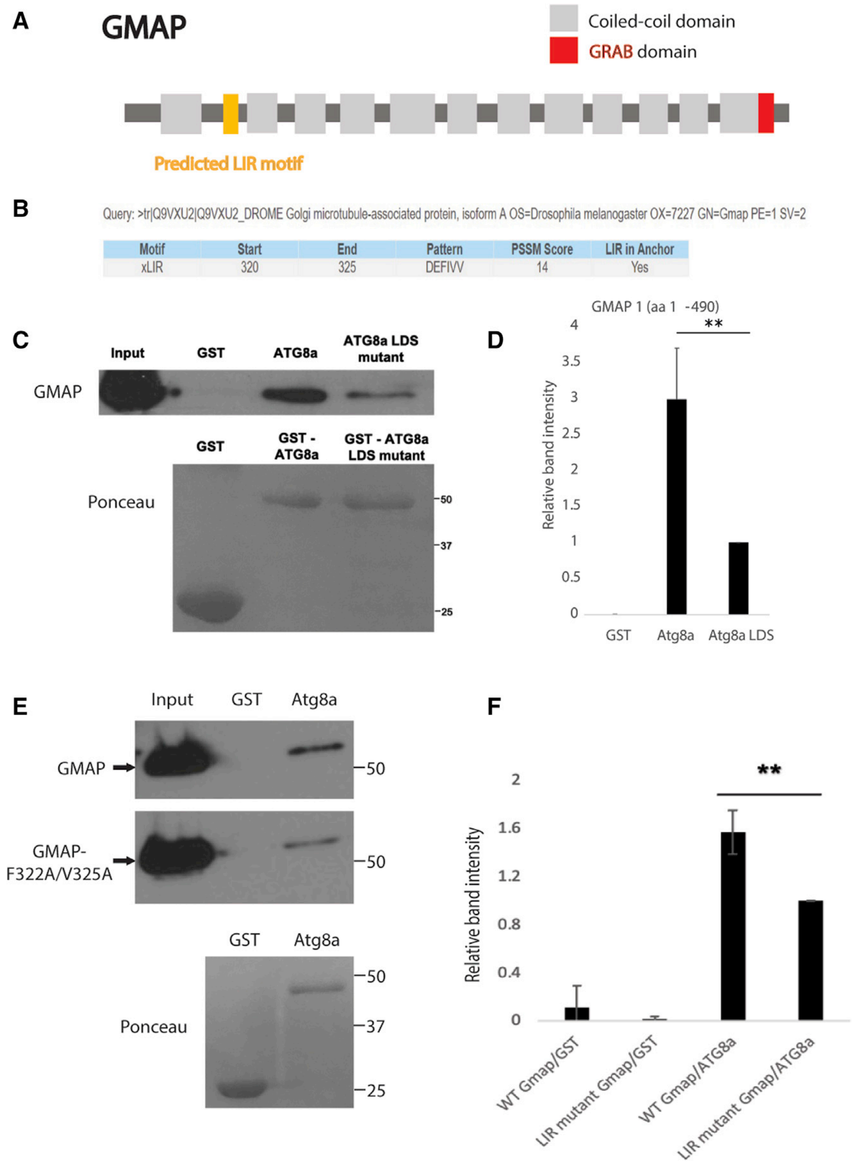

Figure 4. GMAP interacts with Atg8a via a LIR motif

(A) Structure of GMAP. GMAP is a coiled-coil protein which has 12 coiled-coil domains (gray) and a GRAB domain (red). Yellow represents the predicted LIR motif.

(B) GMAP has a predicted LIR motif at position 320–325.

(C and D) GST-pulldown assay between GST-tagged Atg8a-WT or GST-tagged Atg8a-LDS mutant and His-tagged GMAP. GMAP interacts with Atg8a-WT but significantly less with Atg8a-LDS. GST was used as negative control.

(E and F) GST-pulldown assay between GST-tagged Atg8a-WT and His-GMAP or His-GMAP LIR mutant. GMAP interacts with Atg8a. Point mutations of the GMAP LIR motif in positions 322 and 325 by alanine substitutions of the aromatic and hydrophobic residues (F322A and V325A) significantly reduced its binding to Atg8a. GST was used as negative control. A truncated form of GMAP (1–490 aa) was used. Bar charts show means ± SD. Statistical significance was determined using two-tailed Student's t test. **$p < 0.01$. Number of biological repeats (N = 3 for all figures).

(Figure 5B). In addition, knockdown of GMAP led to accumulation of GM130 (Figure 5C). To elucidate the role of GMAP in Golgiphagy we used CRISPR-Cas9 technology to generate GMAP LIR mutants (GMAP F322A V325A) (Figures S2A). GMAP$^{F322A/V325A}$ mutants are homozygous viable. GMAP colocalization with Atg8a during starvation is significantly decreased in GMAP$^{F322A/V325A}$ mutants (Figure 5A). To examine whether GMAP$^{F322A/V325A}$ mutant flies accumulate Golgi markers, we used western blotting. We observed that GM130 significantly accumulates in GMAP LIR mutant flies (Figure 5D). We further used immunofluorescence confocal microscopy to examine the morphology of the Golgi of GMAP$^{F322A/V325A}$ and Atg8a ^{KG07569} flies using GM130 immunostaining. We observed that Golgi appeared to be deformed and elongated compared with wild-type flies (Figures 5E, 5F, and S2B). To further examine Golgi morphology, we used transmission electron microscopy.

We observed that the area and length of Golgi compartments is significantly larger in GMAP$^{F322A/V325A}$ mutants compared with controls (Figure S3). All together, these results suggest that GMAP regulates Golgi complex turnover via selective autophagy.

To examine the role of Atg8aK48A/Y49A and GMAPF322A/V325A mutation in Golgi complex function, we monitored the release of the Glue-Red reporter from the salivary glands of late L3 larvae (Costantino et al., 2008; Csizmadia et al., 2018).

Figure 3. Accumulation of GMAP in Atg8a^{KG07569} and Atg8a$^{K48A/Y49A}$ mutant flies

(A) Western blot analysis shows that GMAP is accumulated in both Atg8a ^{KG07569} and Atg8a$^{K48A/Y49A}$ mutant flies.

(B) Quantification of GMAP in Atg8a^{KG07569} and Atg8a$^{K48A/Y49A}$ mutant flies.

(C) Confocal images from 2-week-old adult brains, GMAP (green) (arrows) and ubiquitin (red) aggregates can be seen in Atg8a^{KG07569} and Atg8a$^{K48A/Y49A}$ mutant flies. DNA was dyed with Hoechst (blue). Scale bars, 10 μm.

(D) Average GMAP puncta size is larger in Atg8a^{KG07569} and Atg8a$^{K48A/Y49A}$ mutant flies compared with wild-type flies. Bar charts show means ± SD. Statistical significance was determined using two-tailed Student's t test. *$p < 0.05$. Number of biological repeats (N = 3 for all figures). Genotypes for all figures: wild-type: w^{1118}/Y, Atg8a LDS: Atg8a $^{K48A/Y49A}$/Y, Atg8a: Atg8a ^{KG07569}/Y.

CellPress
OPEN ACCESS

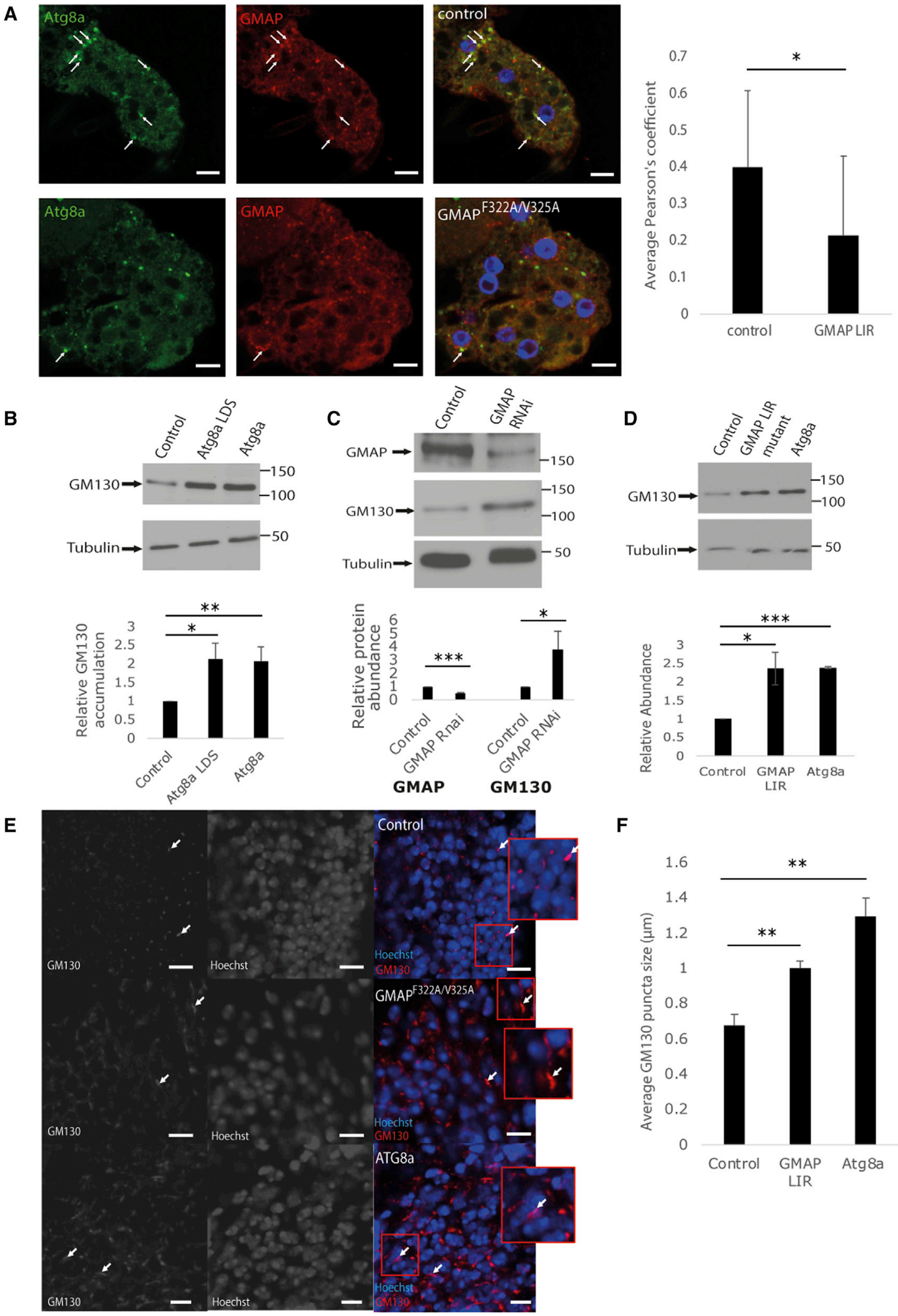

(legend on next page)

As shown in Figure S4, we did not observe secretory defects (Figure S4). To confirm these results, we also investigated the possible retention of the collagen reporter Vkg:GFP (Morin et al., 2001) and the secr-GFP reporter (Pfeiffer et al., 2000), both of which have been used previously to show defects in the secretory machinery (Ke et al., 2018; Zang et al., 2015). Similarly, we found no notable secretion defects (Figure S5).

DISCUSSION

Molecular mechanisms of selective autophagy are mostly characterized in mammals (Johansen and Lamark, 2020). To investigate selective autophagy in the fruit fly *Drosophila melanogaster*, we created Atg8a LDS mutants that cannot bind to LIRCPs. Atg8a LDS mutants have a similar phenotype with Atg8a^{KG07569} mutants (that do not express Atg8a protein): (1) they are viable, (2) they accumulate experimentally verified LIRCPs and ubiquitinated proteins, and (3) and they have reduced lifespan. These results show that LIR motif/LDS interaction is important for the function of Atg8a in autophagy *in vivo*. The accumulation of LIRCPs and ubiquitinated proteins is milder in Atg8a LDS mutants compared with Atg8a^{KG07569} mutants. We speculate that this could be related to a UIM docking site (UDS) that it is not mutated in Atg8a LDS mutants and could contribute to degradation of autophagic substrates. The moderate increase in bulk autophagy observed in Atg8a LDS mutants could be related to stabilization of Atg8a LDS mutant protein, since it is not consumed by LDS binding proteins being degraded by autophagy (like Ref(2)P).

Selective autophagy of mitochondria, peroxisomes, lysosomes, and ER has been described and their receptors have been identified (Anding and Baehrecke, 2017; Johansen and Lamark, 2020). Golgi turnover by autophagy is poorly described. Recently, work by the Johansen group identified CALCOCO1 as a selective autophagy receptor for Golgiphagy (Nthiga et al., 2021). They showed that CALCOCO1 binds the Golgi palmitoyl-transferase ZDHHC17 to mediate Golgi degradation by autophagy during starvation. Depletion of CALCOCO1 causes expansion of the Golgi and accumulation of its proteins. Here, we show that, in *Drosophila*, *cis*-Golgi protein GMAP binds directly to Atg8a (without the involvement of an intermediate receptor) to mediate Golgi turnover and control the size and morphology of the Golgi complex. GMAP binding to Atg8a is mediated by a LIR motif. The observation that interaction of GMAP with Atg8a is not completely abolished by Atg8a$^{K48A/Y49A}$ or GMAP$^{F322A/V325A}$ mutations could suggest the presence of additional LIR motifs or binding domains.

GMAP$^{F322A/V325A}$ mutants exhibit accumulation of *cis*-Golgi markers and elongated Golgi morphology, suggesting a role of GMAP in Golgi turnover. The observations that Atg8a$^{K48A/Y49A}$ or GMAP$^{F322A/V325A}$ mutations did not affect secretion of known secreted proteins suggest that Atg8a and GMAP's LIR motif are not absolutely essential for Golgi secretory function and there may be a redundancy with other proteins (yet to be identified) in the regulation of Golgi complex turnover in relation to its secretory function.

In summary, we have shown that the LDS binding pocket in Atg8a plays an important role in the execution of selective autophagy. We identified the *cis*-Golgi protein GMAP as an Atg8a-interacting protein. We suggest that GMAP mediates Golgi turnover via its LIR motif-mediated interaction with Atg8a. Our study highlights the physiological importance of Atg8a's LDS binding pocket and opens new avenues in the regulation of Golgi turnover by selective autophagy.

Limitations of the study

GMAP was identified as an Atg8a-interacting protein in a proteomics screening in Atg8a LDS mutants. We also identified several other proteins that accumulate in Atg8a LDS mutants. However, our proteomics analysis might not have identified all accumulating proteins. In addition, this study does not characterize other putative docking sites in Atg8a protein, such as the UDS.

STAR★METHODS

Detailed methods are provided in the online version of this paper and include the following:

- KEY RESOURCES TABLE
- RESOURCE AVAILABILITY
  - Lead contact
  - Materials availability
  - Data and code availability
- EXPERIMENTAL MODEL AND SUBJECT DETAILS
  - Fly husbandry and generation of transgenic lines
- METHOD DETAILS
  - Protein extraction and western blotting
  - Immunohistochemistry
  - Proteomics
  - Lifespan measurement
  - Plasmid constructs
  - GST pull-down assays

Figure 5. GMAP regulates Golgi turnover via autophagy

(A) Confocal images showing co-localization of endogenous Atg8a and the GMAP under starvation conditions in adult fat body in control and GMAP $^{F322A/V325A}$ mutant flies.

(B) Western blots showing accumulation of the Golgi marker GM130 in Atg8a ^{KG07569} and Atg8a$^{K48A/Y49A}$ mutant flies compared with wild-type flies.

(C) Western blots showing accumulation of GM130 in GMAP-RNAi lines compared with control RNAi and its quantification shown below.

(D) Western blots showing accumulation of GM130 in GMAP $^{F322A/V325A}$ mutant flies compared with wild-type flies.

(E) Immunofluorescence confocal microscopy of *Drosophila* brain showing increased accumulation of the *cis*-Golgi marker (GM130) and the altered morphology of Golgi in GMAP $^{F322A/V325A}$ and Atg8a ^{KG07569} mutant flies. Scale bars, 10 μm.

(F) Average GM130 puncta size is larger in GMAP $^{F322A/V325A}$ and Atg8a ^{KG07569} mutant flies compared with wild-type flies. Bar charts show means ± SD. Statistical significance was determined using two-tailed Student's t test. *p < 0.05, **p < 0.01, ***p < 0.001. Number of biological repeats (N = 3 for all figures).

Genotypes: (A) Control: w^{1118}/Y, GMAP: GMAP $^{F322A/V325A}$/Y. (B) Control: w^{1118}/Y, Atg8a LDS: Atg8a $^{K48A/Y49A}$/Y, Atg8a: Atg8a ^{KG07569}/Y. (C) Control: yw^{1118};P{attP,y[+],w[3']}/+;da-GAL4/+, GMAP-RNAi: GMAP-RNAi/+; da-GAL4/+. (D and E) Control:w^{1118}/Y, GMAP: GMAP $^{F322A/V325A}$/Y, Atg8a: Atg8a ^{KG07569}/Y.

○ Transmission electron microscopy
- QUANTIFICATION AND STATISTICAL ANALYSIS
  ○ Quantification of transmission electron microscopy
  ○ Quantification of western blots
  ○ Quantification of immunohistochemistry

SUPPLEMENTAL INFORMATION

Supplemental information can be found online at https://doi.org/10.1016/j.celrep.2022.110903.

ACKNOWLEDGMENTS

We thank Prof. P. Therond for the anti-GMAP antibody and Prof. N. Silverman for the anti-Kenny antibody. We thank Prof. Terje Johansen for helpful discussions. We acknowledge WellGenetics Inc. for the construction of Atg8a-LDS and GMAP LIR CRISPR mutants. We thank Warwick Proteomics research technology platform for help with the proteomics analysis and members of Nezis lab for helpful discussions. This work was supported by BBSRC grants BB/L006324/1, BB/P007856/1, BB/V014838/1, and Leverhulme Trust Project grant RPG-2017-023 awarded to I.P.N., and the National Research Development and Innovation Office (KKP 129797 and the National Laboratory for Biotechnology program) awarded to G.J. A.R. and R.G. hold PhD studentships funded by the Midlands Integrative Biosciences Training Partnership funded by BBSRC. P.L. was funded by the Hungarian Academy of Sciences (BO-00514-21-8) and the National Research Development and Innovation Office (FK 138851, ÚNKP-21-5-ELTE-1122).

AUTHOR CONTRIBUTIONS

I.P.N. conceived the project. A.R. performed all the experiments. Y.Z. performed the mass spectrometry analysis. R.G. performed western blotting and immunostainings. P.L. and G.J. performed EM analysis, A.N. and G.C. performed secretion analysis and immunostainings. I.P.N. composed the manuscript. All authors reviewed the manuscript and discussed the work.

DECLARATION OF INTERESTS

The authors declare no competing interests.

Received: September 2, 2021
Revised: March 11, 2022
Accepted: May 11, 2022
Published: May 31, 2022

REFERENCES

Anding, A.L., and Baehrecke, E.H. (2017). Cleaning house: selective autophagy of organelles. Dev. Cell *41*, 10–22. https://doi.org/10.1016/j.devcel.2017.02.016.

Costantino, B.F.B., Bricker, D.K., Alexandre, K., Shen, K., Merriam, J.R., Antoniewski, C., Callender, J.L., Henrich, V.C., Presente, A., and Andres, A.J. (2008). A novel ecdysone receptor mediates steroid-regulated developmental events during the mid-third instar of Drosophila. PLoS Genet. *4*, e1000102. https://doi.org/10.1371/journal.pgen.1000102.

Csizmadia, T., Lőrincz, P., Hegedűs, K., Széplaki, S., Lőw, P., and Juhász, G. (2018). Molecular mechanisms of developmentally programmed crinophagy in Drosophila. J. Cell Biol. *217*, 361–374. https://doi.org/10.1083/jcb.201702145.

De Tito, S., Hervás, J.H., van Vliet, A.R., and Tooze, S.A. (2020). The Golgi as an assembly line to the autophagosome. Trends Biochem. Sci. *45*, 484–496. https://doi.org/10.1016/j.tibs.2020.03.010.

Friggi-Grelin, F., Rabouille, C., and Therond, P. (2006). The cis-Golgi Drosophila GMAP has a role in anterograde transport and Golgi organization *in vivo*, similar to its mammalian ortholog in tissue culture cells. Eur. J. Cell Biol. *85*, 1155–1166. https://doi.org/10.1016/j.ejcb.2006.07.001.

Ichimura, Y., Kumanomidou, T., Sou, Y.S., Mizushima, T., Ezaki, J., Ueno, T., Kominami, E., Yamane, T., Tanaka, K., and Komatsu, M. (2008). Structural basis for sorting mechanism of p62 in selective autophagy. J. Biol. Chem. *283*, 22847–22857. https://doi.org/10.1074/jbc.m802182200.

Jacomin, A.C., Samavedam, S., Promponas, V., and Nezis, I.P. (2016). iLIR database: a web resource for LIR motif-containing proteins in eukaryotes. Autophagy *12*, 1945–1953. https://doi.org/10.1080/15548627.2016.1207016.

Jain, A., Rusten, T.E., Katheder, N., Elvenes, J., Bruun, J.A., Sjøttem, E., Lamark, T., and Johansen, T. (2015). p62/Sequestosome-1, autophagy-related gene 8, and autophagy in Drosophila are regulated by nuclear factor erythroid 2-related factor 2 (NRF2), independent of transcription factor TFEB. J. Biol. Chem. *290*, 14945–14962. https://doi.org/10.1074/jbc.m115.656116.

Jipa, A., Vedelek, V., Merényi, Z., Ürmösi, A., Takáts, S., Kovács, A.L., Horváth, G.V., Sinka, R., and Juhász, G. (2021). Analysis of Drosophila Atg8 proteins reveals multiple lipidation-independent roles. Autophagy *17*, 2565–2575. https://doi.org/10.1080/15548627.2020.1856494.

Johansen, T., and Lamark, T. (2020). Selective autophagy: ATG8 family proteins, LIR motifs and cargo receptors. J. Mol. Biol. *432*, 80–103. https://doi.org/10.1016/j.jmb.2019.07.016.

Kalvari, I., Tsompanis, S., Mulakkal, N.C., Osgood, R., Johansen, T., Nezis, I.P., and Promponas, V.J. (2014). iLIR: a web resource for prediction of Atg8-family interacting proteins. Autophagy *10*, 913–925. https://doi.org/10.4161/auto.28260.

Ke, H., Feng, Z., Liu, M., Sun, T., Dai, J., Ma, M., Liu, L.P., Ni, J.Q., and Pastor-Pareja, J.C. (2018). Collagen secretion screening in Drosophila supports a common secretory machinery and multiple Rab requirements. J Genet Genomics *45*, 299–313. https://doi.org/10.1016/j.jgg.2018.05.002.

Kondo, S., and Ueda, R. (2013). Highly improved gene targeting by germline-specific Cas9 expression in Drosophila. Genetics *195*, 715–721. https://doi.org/10.1534/genetics.113.156737.

Lamb, C.A., Yoshimori, T., and Tooze, S.A. (2013). The autophagosome: origins unknown, biogenesis complex. Nat. Rev. Mol. Cell Biol. *14*, 759–774. https://doi.org/10.1038/nrm3696.

Lu, L.Q., Tang, M.Z., Qi, Z.H., Huang, S.F., He, Y.Q., Li, D.K., Li, L.F., and Chen, L.X. (2020). Regulation of the Golgi apparatus via GOLPH3-mediated new selective autophagy. Life Sci. *253*, 117700. https://doi.org/10.1016/j.lfs.2020.117700.

Marshall, R.S., Hua, Z., Mali, S., McLoughlin, F., and Vierstra, R.D. (2019). ATG8-Binding UIM proteins define a new class of autophagy adaptors and receptors. Cell *177*, 766–781.e24. https://doi.org/10.1016/j.cell.2019.02.009.

Mijaljica, D., Prescott, M., and Devenish, R.J. (2006). Endoplasmic reticulum and Golgi complex: contributions to, and turnover by, autophagy. Traffic *7*, 1590–1595. https://doi.org/10.1111/j.1600-0854.2006.00495.x.

Morin, X., Daneman, R., Zavortink, M., and Chia, W. (2001). A protein trap strategy to detect GFP-tagged proteins expressed from their endogenous loci in *Drosophila*. Proc. Natl. Acad. Sci. U S A *98*, 15050–15055. https://doi.org/10.1073/pnas.261408198.

Nezis, I.P., Simonsen, A., Sagona, A.P., Finley, K., Gaumer, S., Contamine, D., Rusten, T.E., Stenmark, H., and Brech, A. (2008). Ref(2)P, the Drosophila melanogaster homologue of mammalian p62, is required for the formation of protein aggregates in adult brain. J. Cell Biol. *180*, 1065–1071. https://doi.org/10.1083/jcb.200711108.

Noda, N.N., Ohsumi, Y., and Inagaki, F. (2010). Atg8-family interacting motif crucial for selective autophagy. FEBS Lett. *584*, 1379–1385. https://doi.org/10.1016/j.febslet.2010.01.018.

Nthiga, T.M., Shrestha, B.K., Bruun, J.A., Larsen, K.B., Lamark, T., and Johansen, T. (2021). Regulation of Golgi turnover by CALCOCO1-mediated selective autophagy. J. Cell Biol. *220*, e202006128. https://doi.org/10.1083/jcb.202006128.

Pankiv, S., Clausen, T.H., Lamark, T., Brech, A., Bruun, J.A., Outzen, H., Overvatn, A., Bjorkoy, G., and Johansen, T. (2007). p62/SQSTM1 binds directly to Atg8/LC3 to facilitate degradation of ubiquitinated protein aggregates by

autophagy. J. Biol. Chem. *282*, 24131–24145. https://doi.org/10.1074/jbc.m702824200.

Pfeiffer, S., Alexandre, C., Calleja, M., and Vincent, J.P. (2000). The progeny of wingless-expressing cells deliver the signal at a distance in Drosophila embryos. Curr. Biol. *10*, 321–324. https://doi.org/10.1016/s0960-9822(00)00381-x.

Randow, F., and Youle, R.J. (2014). Self and nonself: how autophagy targets mitochondria and bacteria. Cell Host Microbe *15*, 403–411. https://doi.org/10.1016/j.chom.2014.03.012.

Rogov, V.V., Stolz, A., Ravichandran, A.C., Rios-Szwed, D.O., Suzuki, H., Kniss, A., Löhr, F., Wakatsuki, S., Dötsch, V., Dikic, I., et al. (2017). Structural and functional analysis of the GABARAP interaction motif (GIM). EMBO Rep. *18*, 1382–1396. https://doi.org/10.15252/embr.201643587.

Scott, R.C., Juhász, G., and Neufeld, T.P. (2007). Direct induction of autophagy by Atg1 inhibits cell growth and induces apoptotic cell death. Curr. Biol. *17*, 1–11.

Sinka, R., Gillingham, A.K., Kondylis, V., and Munro, S. (2008). Golgi coiled-coil proteins contain multiple binding sites for Rab family G proteins. J. Cell Biol. *183*, 607–615. https://doi.org/10.1083/jcb.200808018.

Tusco, R., Jacomin, A.C., Jain, A., Penman, B.S., Larsen, K.B., Johansen, T., and Nezis, I.P. (2017). Kenny mediates selective autophagic degradation of the IKK complex to control innate immune responses. Nat. Commun. *8*, 1264. https://doi.org/10.1038/s41467-017-01287-9.

Wirth, M., Zhang, W., Razi, M., Nyoni, L., Joshi, D., O'Reilly, N., Johansen, T., Tooze, S.A., and Mouilleron, S. (2019). Molecular determinants regulating selective binding of autophagy adapters and receptors to ATG8 proteins. Nat Commun. *10*, 2055. https://doi.org/10.1038/s41467-019-10059-6.

Zang, Y., Wan, M., Liu, M., Ke, H., Ma, S., Liu, L.P., Ni, J.Q., and Carlos Pastor-Pareja, J. (2015). Plasma membrane overgrowth causes fibrotic collagen accumulation and immune activation in Drosophila adipocytes. Elife *4*, e07187. https://doi.org/10.7554/elife.07187.

STAR★METHODS

KEY RESOURCES TABLE

REAGENT or RESOURCE	SOURCE	IDENTIFIER
Antibodies		
Rabbit anti-Ref(2)P	Abcam	Cat#ab178440
anti-Kenny	Gift from Dr N. Silverman	N/A
Mouse anti-mono/poly-ubiquitinated proteins (FK2)	Enzo	Cat#BML-PW8810-0100
Rabbit anti-GM130	Abcam	Cat#ab30637
Mouse anti-6xHis tag®	Abcam	Cat#ab18184
Rabbit anti-dGMAP (Western blotting)	Gift from Dr Pascal Therond	N/A
Goat anti-GMAP (IF)	Developmental Studies Hybridoma Bank	RRID:AB_2618259
Mouse anti-alpha tubulin	Sigma-Aldrich	Cat#T5168; RRID:AB_477579
Rabbit anti-Gabarap+GabarapL1+GabarapL2	Abcam	Cat#ab109364
Rabbit Anti-Mouse IgG HRP	Thermo Fisher Scientific	Cat#31450; RRID:AB_228427
Goat Anti-Rabbit IgG HRP	Thermo Fisher Scientific	Cat#31460; RRID:AB_228341
Rabbit Anti-Goat IgG CF488A	Sigma-Aldrich	Cat#SAB4600053
Goat Anti-Mouse IgG CF488A	Sigma-Aldrich	Cat#SAB4600042
Chicken Anti-Goat IgG CF488A	Sigma-Aldrich	Cat#SAB4600028
Goat Anti-Rabbit IgG CF568	Sigma-Aldrich	Cat#SAB4600084
Goat Anti-Mouse IgG CF568	Sigma-Aldrich	Cat#SAB4600082
Donkey Anti-Mouse IgG CF568	Sigma-Aldrich	Cat#SAB4600075
Bacterial and virus strains		
Rosetta™ 2(DE3) Singles™ Competent Cells	Novagen	Cat#71400
Chemicals, peptides, and recombinant proteins		
cOmplete™ ULTRA Tablets, Mini, EDTA-free, *EASYpack* Protease Inhibitor Cocktail	Roche	Cat#5892791001
Glutathione Sepharose® 4B	Sigma-Alrich	Cat#17-0756-01
Formaldehyde	Sigma-Alrich	Cat#F8775
Critical commercial assays		
QuickChange site-directed mutagenesis	Stratagene	200523
BigDye™ Terminator v3.1 Cycle Sequencing Kit	Applied Biosystems	Cat#4337455
Phusion™ High-Fidelity DNA Polymerase	Thermo Fisher Scientific	Cat#F-530XL
Deposited data		
Identified proteins from Atg8aKG07569 mutants, Atg8aK48A/Y49A mutants, and wild-type (WT) flies	This paper	Table S1
Differential protein expression between WT and mutant Drosophila	This paper	Table S2
Experimental models: Organisms/strains		
Drosophila melanogaster: control/WT: w^{1118}/Y	Bloomington *Drosophila* stock center	#3605
Drosophila melanogaster: da-GAL4: w[*]; P{w[+mW.hs]=GAL4-da.G32}UH1, Sb[1]/TM6B, Tb[1]	Bloomington *Drosophila* stock center	#55851
Drosophila melanogaster: Atg8a LDS: $Atg8a^{K48A/Y49A}$/Y	This paper; created by CRISPR-mediated mutagenesis; Well Genetics	N/A
Drosophila melanogaster: Atg8a: $Atg8a^{KG07569}$/Y	Nezis et al. (2008)	N/A

(*Continued on next page*)

Continued		
REAGENT or RESOURCE	SOURCE	IDENTIFIER
Drosophila melanogaster: Control RNAi: y,w^{1118};P{attP,y[+],w[3`]}/+;da-GAL4/+	Vienna *Drosophila* Resource Center	#60100
Drosophila melanogaster: GMAP-RNAi: P{KK107249}VIE-260B	Vienna *Drosophila* Resource Center	#108063
Drosophila melanogaster: GMAP LIR: GMAP$^{F322A/V325A}$/Y	This paper; created by CRISPR-mediated mutagenesis; Well Genetics	N/A
Drosophila melanogaster: secr.EGFP: UAS-secr.EGFP/+; tub-GAL4/+	Gift from Dr Jean-Paul Vincent	N/A
Drosophila melanogaster: Vkg::GFP: P{PTT-un}vkg^{G00454}/+	Gift from Prof József Mihály	N/A
Drosophila melanogaster: P{Sgs3-DsRed}	Gift from Andrew Andres	N/A
Oligonucleotides		
GMAP truncated protein: Forward Primer: CGGAATTCATGTCGTGGCTGAACAGC	This paper	N/A
GMAP truncated protein: Reverse Primer (R490): CCGCTCGAGTTATTAGTCCGCATCGTCCA	This paper	N/A
Primers for GMAP F322A/V325A mutagenesis: Forward GCACAGCGAGGATGAGGCCATAG TTGCACGCCAAGCGGATGCC	This paper	N/A
Primers for GMAP F322A/V325A mutagenesis: Reverse Primer: GGCATCCGCTTGGCGTGCAACT ATGGCCTCATCCTCGCTGTGC	This paper	N/A
Recombinant DNA		
Plasmid: pET28a(+)	Novagen	Cat#69864
Software and algorithms		
iLIR database	Kalvari et al. (2014)	http://repeat.biol.ucy.ac.cy/iLIR/
iTEM software	N/A	https://www.itemsoft.com/

RESOURCE AVAILABILITY

Lead contact

Additional information and requests for reagents and protocols should be directed to and will be fulfilled by the Lead Contact, Ioannis Nezis (I.Nezis@warwick.ac.uk).

Materials availability

All materials are publicly available. Please contact Prof. Ioannis Nezis.

Data and code availability

- All data generated and reported in this paper are available from the lead contact upon request.
- This paper does not report original code.
- Any additional information required to reanalyse the data reported in this paper is available from the lead contact upon request

EXPERIMENTAL MODEL AND SUBJECT DETAILS

Fly husbandry and generation of transgenic lines

Flies used in experiments were kept at 25°C and 70% humidity raised on cornmeal-based feed. Atg8a [Atg8a^{KG07569}] flies were from our lab (Nezis et al., 2008). da-GAL4 (#55851) and w^{1118} control/WT (#3605) flies were obtained from the Bloomington Drosophila stock centre. GMAP-RNAi (#108063) and control RNAi (#60100) flies were obtained from the Vienna Drosophila Resource Center. secr.EGFP flies were gifted by Dr Jean-Paul Vincent and Vkg::GFP flies were gifted by Prof József Mihály. Sgs3-dsRed2 (or simply GlueRed, controlled by genomic *sgs3* promoter) reporter was kindly provided by Andrew Andres, University of Nevada, Las Vegas, NV, USA (Costantino et al., 2008).

CRISPR-mediated mutagenesis to create Atg8a$^{K48A/Y49A}$ and GMAP$^{F322A/V325A}$ mutants was performed by WellGenetics Inc. using modified methods of Kondo and Ueda (2013). In brief, gRNA sequences CGGTCAGGTCGGAAGGCACC[AGG] (for Atg8a) and GTTCATAGTTGTACGCCAAG[CGG] (for GMAP) were cloned into U6 promoter plasmid(s). Cassette K48A/Y49A-PBacDsRed or F322A/V325A-PBacDsRed containing two PiggyBac sites, 3xP3-DsRed, designed point mutation and two 1kb-homology arms were cloned into pUC57-Kan as donor template for repair. The two homology arms of Atg8a/CG32672 or Gmap/CG33206 were amplified by Phusion High-Fidelity DNA Polymerase (Thermo Scientific) from genomic DNA at optimized condition, which reflects sequences of the injection strain *in vivo*. Any mismatches found in coding regions are considered as polymorphisms. The K48A/Y49A-PBacDsRed or F322A/V325A-PBacDsRed cassette was obtained from sequence verified plasmid stock by restriction enzyme digestion. The cassette and two homology arms were cloned by sequence ligation-independent cloning method into vector pUC57-Kan, followed by standard transformation protocol, colony PCR selection and sequencing. Two homology arms and junctions of cassette fragment(s) were confirmed by PCR and sequencing. Atg8a-targeting or GMAP-targeting gRNAs and hs-Cas9 were supplied in DNA plasmids, together with donor plasmid for microinjection into embryos of control strain w1118. F1 flies carrying selection marker of 3xP3-RFP were further validated by genomic PCR and sequencing. CRISPR generates a break in Atg8a or GMAP and is replaced by cassette K48A/Y49A-PBacDsRed or F322A/V325A-PBacDsRed respectively. For ATG8a K48A Y49A CRISPR mutants, 13 putative mutants tested and finally 4 independent lines were established. For GMAP F322A V325A CRISPR mutants, 6 putative mutants tested and finally 1 independent line was established.

METHOD DETAILS

Protein extraction and western blotting

Protein content was extracted from the head and the full fly body in RIPA lysis buffer (50 mM Tris pH 7.4, 150 mM NaCl, 1% Igepal, 0.5% sodium deoxycholate, 0.1% SDS supplemented with cOmplete^{TM} ULTRA EDTA-free protease inhibitor cocktail (Roche, #5892791001)) using a motorized mortar and pestle. Protein concentrations were determined by the Bradford method. 100–200 μg proteins were loaded on polyacrylamide gels and were transferred onto PVDF membranes (cold wet transfer in 10% ethanol for 1 h at 100 V). Membranes were blocked in 5% non-fat milk in TBST (0.1% Tween-20 in TBS) for 1 h. Primary antibodies diluted in TBST were incubated overnight at 4C with gentle agitation. HRP-coupled secondary antibodies binding was done at room temperature (RT) for 1 h in 1% non-fat milk dissolved in TBST and ECL mix incubation for 2 min. All washes were performed for 10 min in TBST at RT.

Immunohistochemistry

Fly tissues were dissected in PBS and fixed for 30 min in 4% formaldehyde (#F8775) in PBS. Blocking and antibody incubations were performed in PBT (0.3% BSA, 0.3% Triton X-100 in PBS). Primary antibodies were incubated overnight at 4°C in PBST, secondary antibodies were incubated 2 h at room temperature in PBST. Samples were observed under a Zeiss 880 confocal microscope and an ApoTome 2-fitted Zeiss AxioImager M.2 microscope.

Proteomics

Proteins were extracted from the drosophila head by using RIPA buffer. Solubilized proteins were recovered by centrifugation (12,000 g, 10 min) and placed in an ultrafiltration tube (MWCO 3,000, Millipore, USA), and reduced with 15 mM dithiothreitol for 120 min, and alkylated with 50 mM iodoacetamide for 60 min in the dark. Protein samples were washed three times with 50 mM NH4HCO3 and then digested with trypsin at a weight ratio of 1:50 (trypsin:protein) for 20 h at 37°C. Tryptic peptides were recovered by centrifugation, lyophilized, and resuspended in 40 μL 0.1% formic acid. Tryptic peptides (4 μL) were separated on a Thermo Fisher Scientific EASY-nLC 1000 system using a Thermo Fisher Scientific EASY-Spray column (C18, 2 μm, 100 Å, 50 μm × 15 cm), and were analyzed using a Thermo Scientific Q Exactive mass spectrometer. Four biological replicates were used for the LC-MS/MS analyses. Mass spectra raw data were analysed using MaxQuant software. Peptide searches were performed with Andromeda search algorithms. All common contaminants and reverse hits were removed. The label-free intensity quantification (LFQ) algorithm in MaxQuant was used to estimates the protein abundance. Identified proteins were listed in Table S1. FactorMineR was used to perform PCA analysis on the basis of protein expression (log10-transformed LFQ values). To find the differential proteins between wild-type and mutant drosophila, unpaired *t*-tests were used with a significance level set at $p < 0.05$ and a cut-off difference of more than two-fold (Table S2). LIR motifs were predicted using iLIR software at https://ilir.warwick.ac.uk.

Lifespan measurement

Fly lines used were isogenic. Atg8a LDS (Atg8a$^{K48A/Y49A}$) and Atg8a (Atg8a^{KG07569}, (Scott et al., 2007)) mutants were backcrossed to the w^{1118} strain for at least 6 generations to produce isogenic lines. We used the Kaplan-Meier method to measure lifespan of flies, which estimates survival probability of each risk group according to daily death events counted. Male and female flies were collected within 24 h from hatching and cohorts of 20–25 flies were maintained on standard Drosophila food at 25°C in a humidified incubator. Flies were transferred into new tubes every 2–3 days. Dead events were recorded daily. Atg8a LDS mutants eclose at the same frequency as background matched controls. Survival curves were constructed in Prism (GraphPad, versions 8 and 9), which was also used to perform the statistical analysis for curve comparison using the Mantel-Cox test.

Plasmid constructs

GMAP plasmids were obtained from Drosophila Genomics Resource Centre. Sequences of the GMAP were amplified by PCR and inserted in desired plasmid using either Gateway recombination system or restriction enzyme cloning. PCR products were amplified from cDNA using Phusion high fidelity DNA polymerase with primers containing the Gateway recombination site or restriction enzyme sites for Gateway entry vector and cloned into pDONR221 or pENTR using Gateway recombination cloning. Point mutants were generated using the QuickChange site-directed mutagenesis (Stratagene, #200523). Plasmid constructs were verified by conventional restriction enzyme digestion and/or by DNA sequencing (Applied Biosystems, #4337455).

GST pull-down assays

Bait proteins (GST alone and GST-Atg8a) as well as prey proteins (GMAP and GMAP LIR mutants) were expressed in RosettaTM 2(DE3) competent cells (Novagen, #71400). GST pull-down assays were performed using recombinant proteins produced in bacteria. Bait proteins were captured using Glutathione-Sepharose beads (Glutathione Sepharose® 4B (Sigma-Aldrich, #17-0756-01)), which were incubated for 40 mins at 4°C. A volume of 10 mL of the *in vitro* translation reaction products (0.5 mg of plasmid in a 25 mL reaction volume) were incubated with 1–10 mg of GST-recombinant protein in 200 mL of NETN buffer (50 mM Tris, pH 8.0, 150 mM NaCl, 1 mM EDTA, 0.5% Nonidet P-40, 1 mM dithiothreitol supplemented with cOmplete^{TM} ULTRA Mini EDTA-free protease inhibitor cocktail (Roche, #5892791001) for 2 h at 4°C, washed six times with 1 mL of NETN buffer, boiled with 2X SDS gel loading buffer, and subjected to SDS-PAGE. Gels were stained with Coomassie Blue and vacuum-dried.

Transmission electron microscopy

Brains of 20 days (after emerging from the pupal case) old adult control and mutant animals were dissected in ice cold PBS, then fixed with 3.2% paraformaldehyde, 1% glutaraldehyde, 1% sucrose, and 3 mM $CaCl_2$ in 0.1 N sodium cacodylate (pH 7.4, overnight, 4°C). Next day samples were washed with sodium cacodylate then post-fixed in 0.5% osmium tetroxide (60 min, RT) then in half-saturated aqueous uranyl acetate (30 min, RT). Samples were then dehydrated in graded series of ethanol and embedded in araldite to the manufacturer's instructions. Ultrathin sections (from 5 control and 5 mutant animals) were stained with Reynold's lead citrate and viewed at 80 kV operating voltage on a JEM-1011 transmission electron microscope (JEOL) equipped with a Morada digital camera (Olympus) using iTEM software (Olympus). All reagents and materials used for electron microscopy were obtained from Merck. The area and width of Golgi apparatuses of cortical neurons of the protocerebrum were measured using iTEM software (Olympus).

QUANTIFICATION AND STATISTICAL ANALYSIS

Quantification of transmission electron microscopy

5 individuals per genotype were analysed (total 10 individuals). Neurons originated from the cortex of the protocerebrum were analysed. Out of hundreds of cells examined, 27 control and 28 GMAP LIR mutant cells contained one "whole" Golgi. The area and width of Golgi apparatuses were measured using iTEM software (Olympus). Cells containing at least three Golgi cisterns were quantified only. Cells in which the Golgi apparatus contained less than three cisterns or contained only vesicles were not evaluated as these Golgi apparatuses were considered partial. The quantified data were evaluated using SPSS21 (IBM) and independent samples u-test.

Quantification of western blots

Statistical analyses were done with Prism6/7 software (GraphPad). Western blot protein bands were quantified using ImageJ/FIJI 2.0 using the Gels tool. A histogram was generated for each band where the peaks were proportional to the intensity of the band. The area under the curve was used as the quantitative value. Where necessary these bands were normalised to control bands. At least three biological repeats were done and averaged. For the comparison of two groups, a two-tailed *t*-test was used.

Quantification of immunohistochemistry

Quantifications in Figures 1D, 1E, 3C, D, 5E, 5F and S2B: For every figure the data are from 5 images per brain, 5 brains per genotype (25 images per genotype). 100 puncta per image were assessed. The measurement of puncta size was done with the line tool in ImageJ/FIJI 2.0.

Quantifications in Figure 5A: wild type:13 fat bodies (1 per fly), 20 images. GMAP LIR: 12 fat bodies (1 per fly), 14 images. Signal colocalization was assessed by Pearson's correlation co-efficient within the set regions of interest (ROIs) which were obtained through the creation of binary masks and background subtracted for the creation of ROIs where appropriate. Counts and puncta size were obtained similarly through the creation of binary masks and the Analyze Particles tool utilised, and counts normalised to the confocal image area in μm^2. Compound figures were assembled in Adobe Illustrator 2022 (version 26.2).

Cell Reports, Volume *39*

Supplemental information

GMAP is an Atg8a-interacting protein that regulates Golgi turnover in *Drosophila*

Ashrafur Rahman, Peter Lőrincz, Raksha Gohel, Anikó Nagy, Gábor Csordás, Yan Zhang, Gábor Juhász, and Ioannis P. Nezis

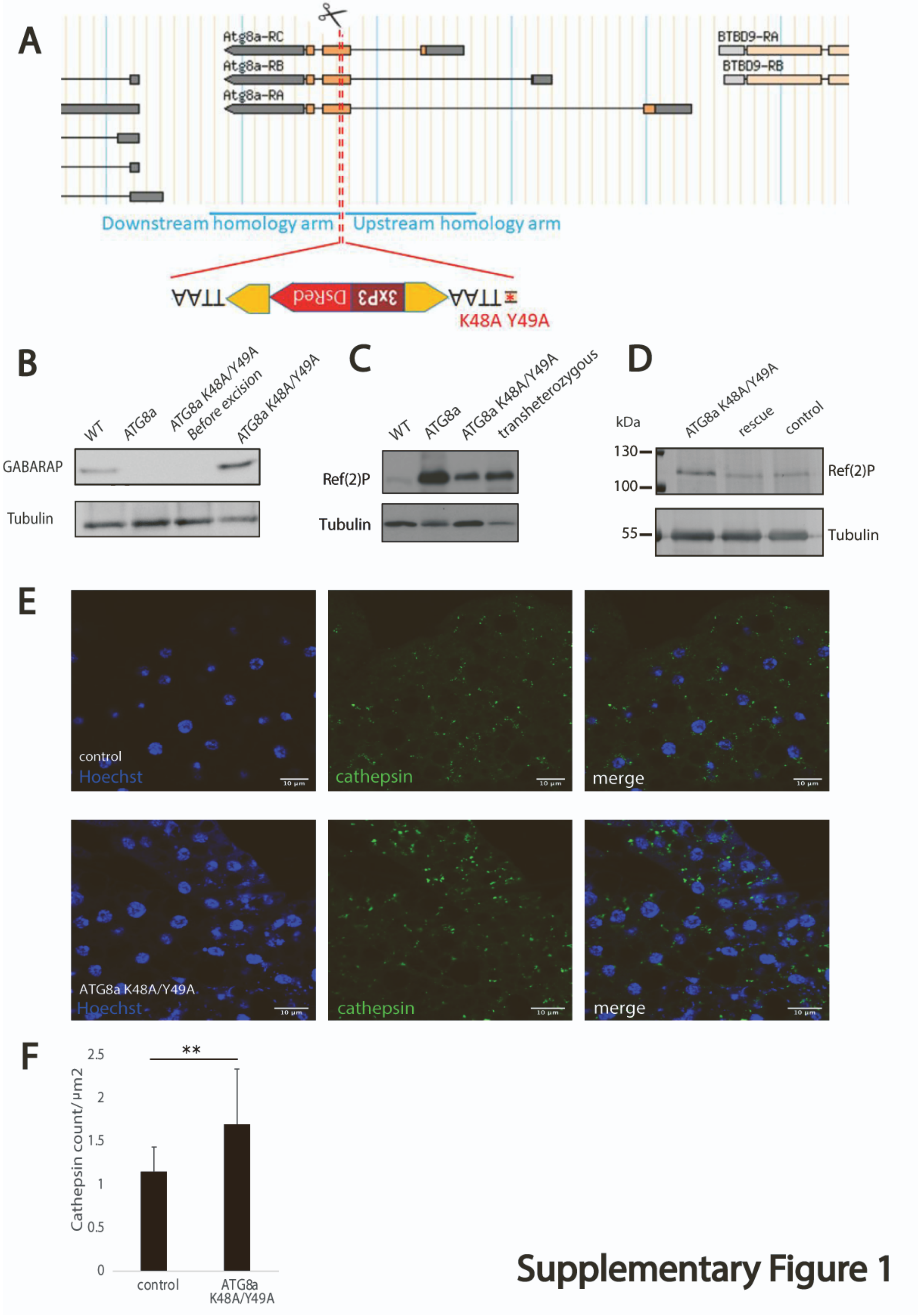

Supplementary Figure 1

Supplementary Figure 1 (Related to Figure 1). Characterization of Atg8a$^{K48A/Y49A}$ mutants.

(A) Schematics showing the generation of Atg8a$^{K48A/Y49A}$ mutant flies using CRISPR. **(B)** Western blots showing expression of Atg8a. Expression of Atg8a protein can be seen in the wild type flies (WT) as well as the Atg8a$^{K48A/Y49A}$ mutants. This shows Atg8a is successfully being expressed in the Atg8a CRISPR mutant flies. Atg8a ^{KG07569} flies were used as a negative control as there was no expression of Atg8a. There is also no expression of Atg8a in the Exon2 line prior to excision which is expected as the selectable marker must first be excised before proper expression can take place. **(C)** Western blots showing Ref(2)P accumulation in Atg8a ^{KG07569}, Atg8a$^{K48A/Y49A}$ and Atg8a ^{KG07569}/Atg8a$^{K48A/Y49A}$ trans-heterozygous flies. **(D)** Western blot showing rescue of Ref(2)P accumulation in Atg8a$^{K48A/Y49A}$ flies with co-expression of 3xmCherry-Atg8a. **(E-F)** Cathepsin L staining of adult fat body in starved wild type and Atg8a$^{K48A/Y49A}$ mutant flies. Number of biological repeats (N)=3 for all figures.

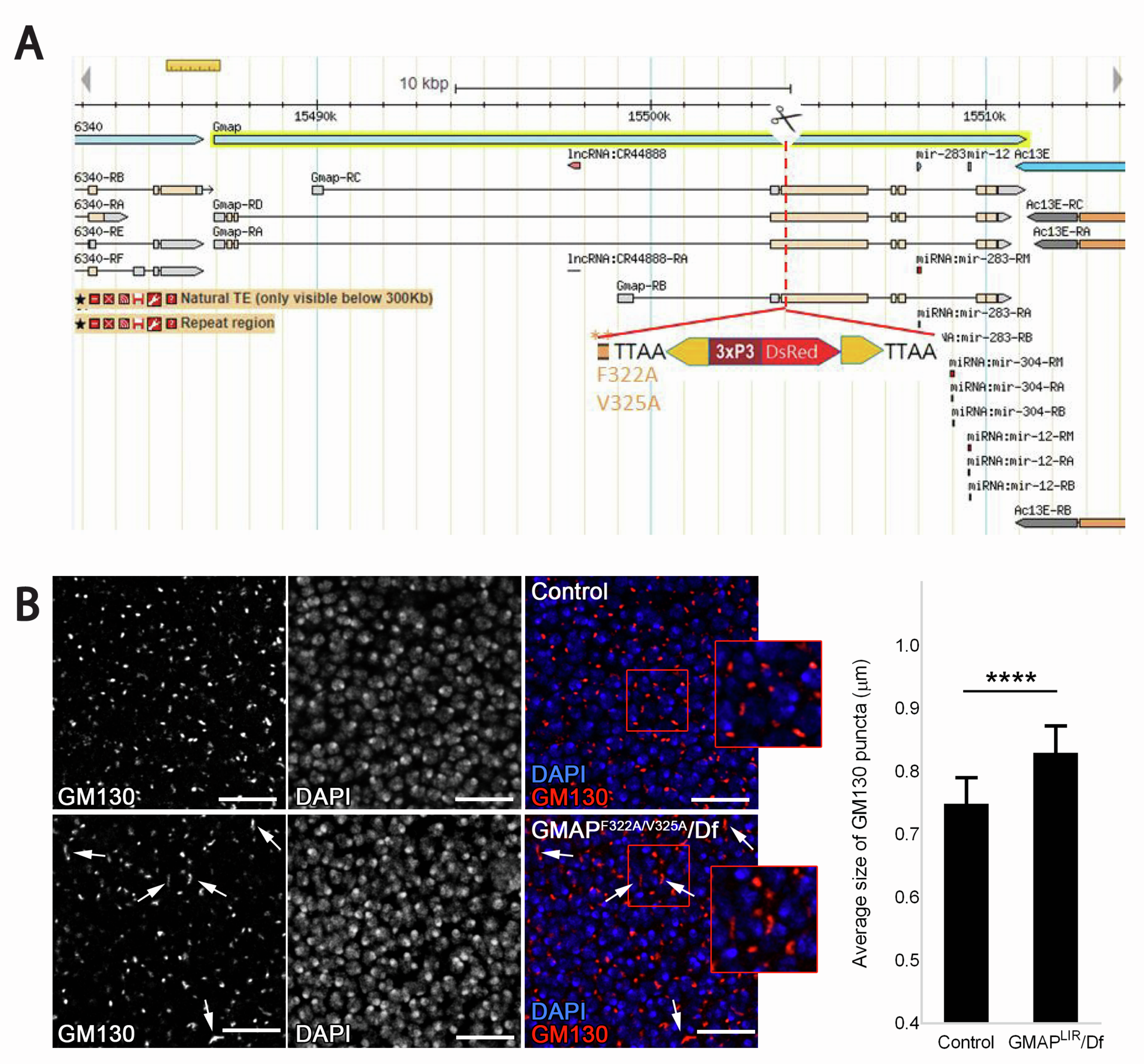
A
10 kbp
15490k
15500k
15510k
6340
Gmap
lncRNA:CR44888
mir-283
mir-12
Ac13E
6340-RB
6340-RA
6340-RE
6340-RF
Gmap-RC
Gmap-RD
Gmap-RA
Gmap-RB
lncRNA:CR44888-RA
Ac13E-RC
Ac13E-RA
Ac13E-RB
miRNA:mir-283-RM
miRNA:mir-283-RA
miRNA:mir-304-RM
miRNA:mir-304-RA
miRNA:mir-304-RB
miRNA:mir-12-RM
miRNA:mir-12-RA
miRNA:mir-12-RB
Natural TE (only visible below 300Kb)
Repeat region
TTAA
3xP3 DsRed
TTAA
F322A
V325A
B
GM130
DAPI
Control
DAPI
GM130
GM130
DAPI
GMAPF322A/V325A/Df
DAPI
GM130
Average size of GM130 puncta (µm)
1.0
0.9
0.8
0.7
0.6
0.5
0.4
****
Control
GMAPLIR/Df

Supplementary Figure 2 (Related to Figure 5). GMAP$^{F322A/V325A}$ mutants exhibit elongated Golgi morphology.

(A) Schematics showing the generation of GMAP$^{F322A/V325A}$ mutant flies using CRISPR. (**B**) Similarly to GMAP$^{F322A/V325A}$ homo/hemizygotes, GMAP $^{F322A/V325A}$ /Df trans-heterozygotes show increased Golgi marker (GM130) accumulation and altered Golgi morphology. Images are single confocal planes. Quantification of average size of GM130 puncta. Average GM130 puncta size is larger in GMAP $^{F322A/V325A}$ /Df brains was significantly higher than in wild type flies. Bar charts show means ± s.d. Statistical significance was determined using two-tailed Student's *t*-test. ****$p < 0.0001$. Number of biological repeats (N)=3 for all figures. Scale bars: 10 μm.

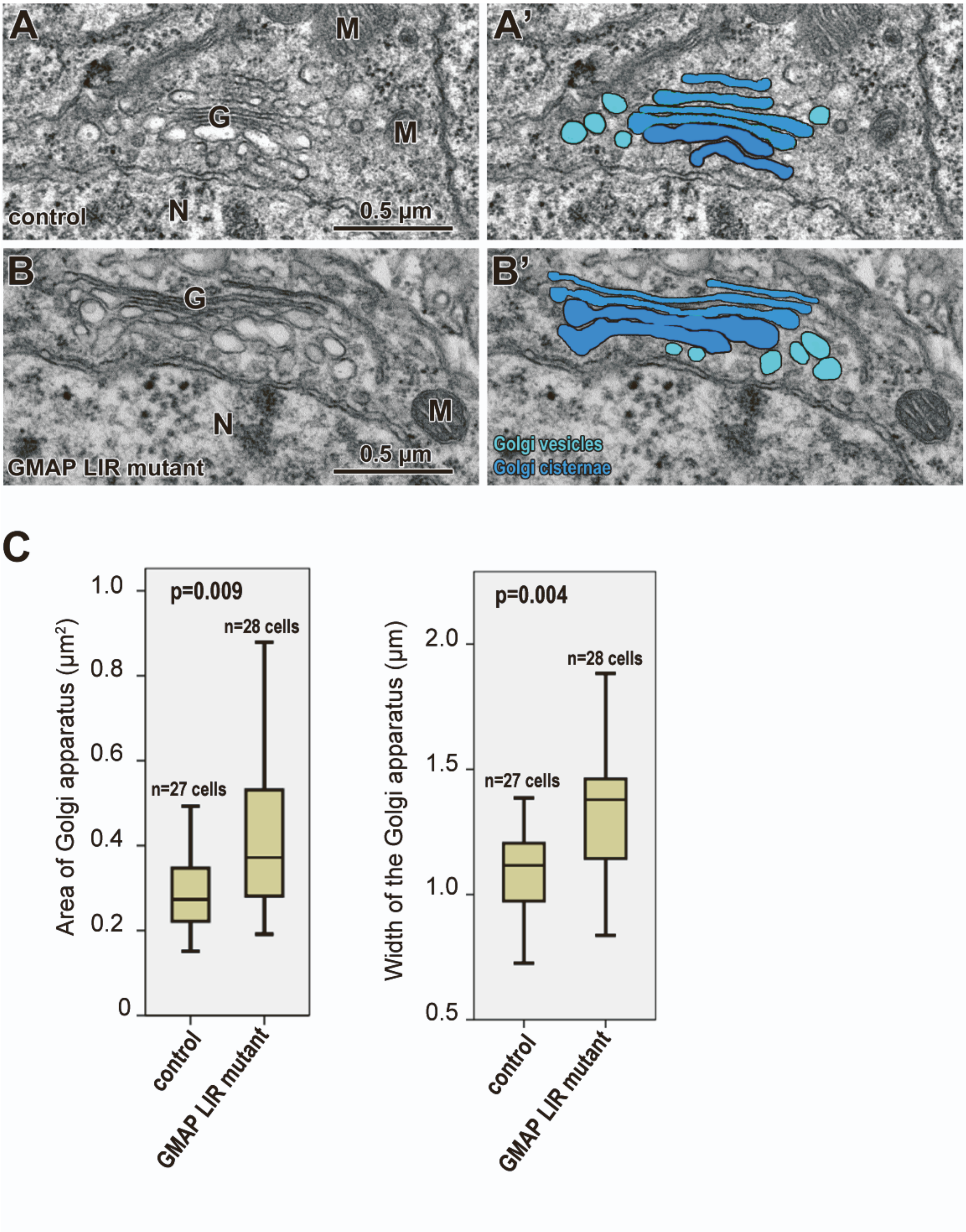

Supplementary Figure 3

Supplementary Figure 3 (Related to Figure 5). GMAP$^{F322A/V325A}$ mutants exhibit elongated Golgi morphology.

Electron micrographs of adult neurons of control **(A)** and GMAP$^{F322A/V325A}$ mutants **(B)**. Enlarged Golgi cisternae are readily apparent in mutants compared to control **(A-C)**. Abbreviations: Golgi (G), Nucleus (N), Mitochondria (M). (N=27 for control and N=28 for mutants). Scale bars: 0.5 µm.

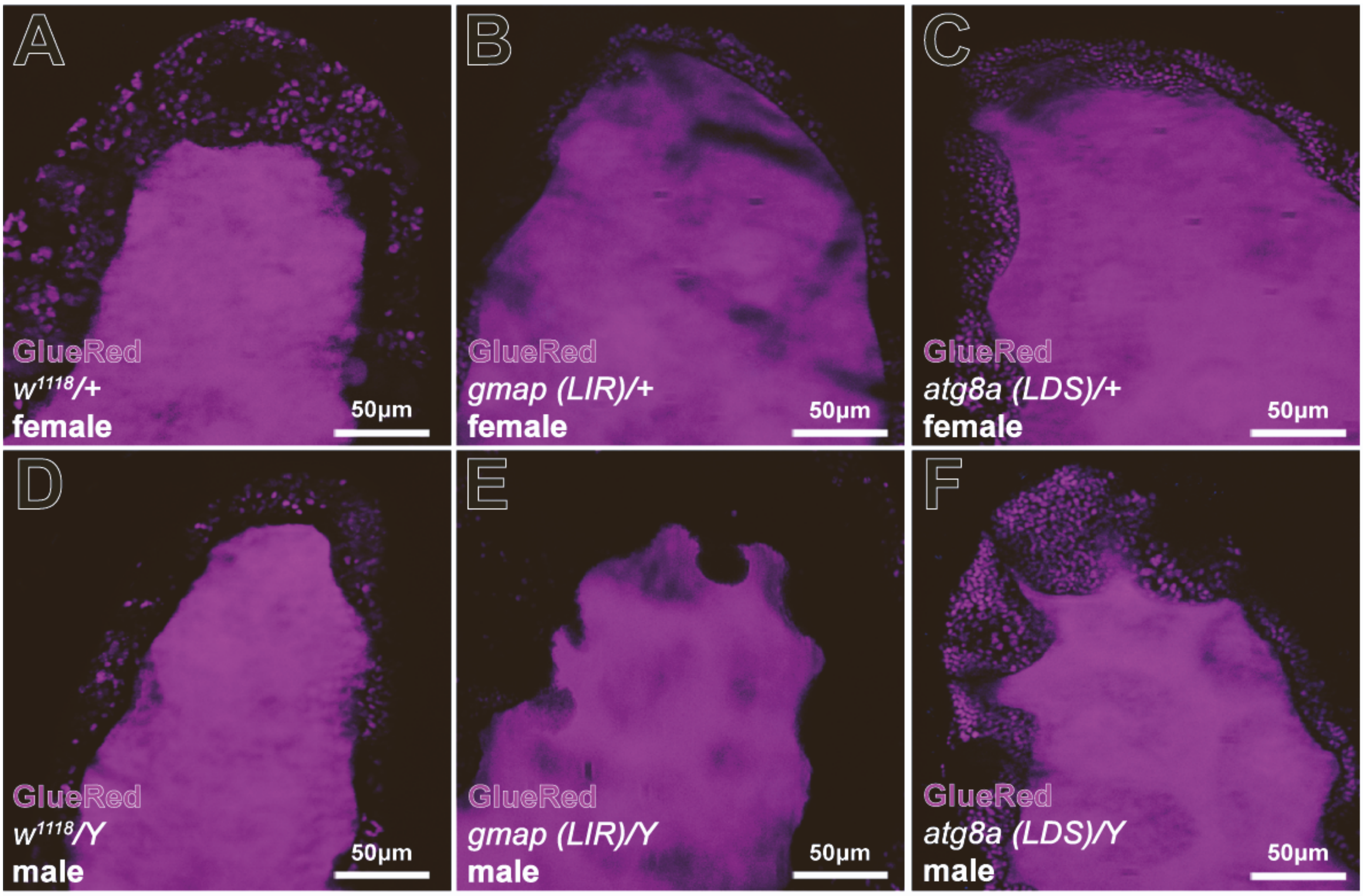

Supplementary Figure 4

Supplementary Figure 4 (Related to Figure 5). GMAP$^{F322A/V325A}$ mutants do not exhibit secretion defects.

The salivary gland-specific (sgs3 promoter-driven) sgs3-DsRed reporter was crossed into the genotypes shown in the images. White prepupal salivary glands are visible in all image panels, and the secreted glue clearly fills the lumen in all cases, with residual granules remaining in cells (but its amount is very variable even in controls). Thus, there is no indication of a secretory defect in GMAP$^{F322A/V325A}$ mutants. Number of biological repeats (N)=3 for all figures.

Genotypes: A: w1118: w^{1118}/+ ; P{Sgs3-DsRed}/+ ; +/+

B: GMAP LIR: GMAP$^{F322A/V325A}$/+; P{Sgs3-DsRed}/+ ; +/+

C: Atg8a LDS: Atg8a$^{K48A/Y49A}$/+; P{Sgs3-DsRed}/+ ; +/+

D: w1118: w^{1118}/Y ; P{Sgs3-DsRed}/+ ; +/+

E: GMAP LIR: GMAP$^{F322A/V325A}$/Y; P{Sgs3-DsRed}/+ ; +/+

F: Atg8a LDS: Atg8a$^{K48A/Y49A}$/Y; P{Sgs3-DsRed}/+ ; +/+

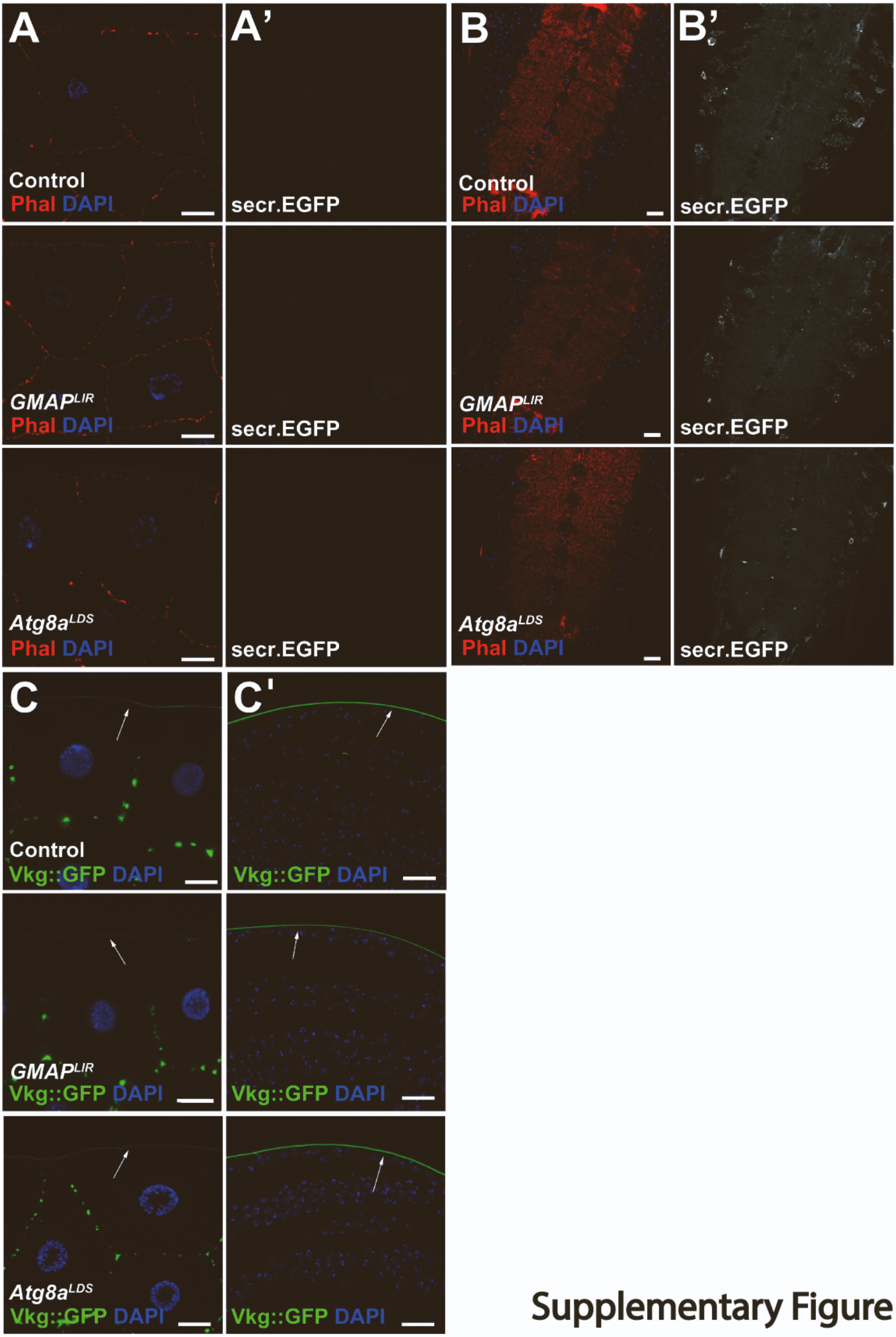

Supplementary Figure 5

Supplementary Figure 5 (Related to Figure 5). GMAP$^{F322A/V325A}$ mutants do not exhibit secretion defects.

(A-B) Fluorescent images show that GMAP $^{F322A/V325A}$ and Atg8a$^{K48A/Y49A}$ mutations do not cause retention of the ubiquitously expressed secr.EGFP secretion reporter in the larval adipose tissue (A) and the larval ventral nerve cord (B). Tissues were counterstained with Phalloidin. **(C)** GMAP $^{F322A/V325A}$ or Atg8a$^{K48A/Y49A}$ did not perturb Collagen IV secretion or caused intracellular retention in the larval adipose tissue (C), or in the wing imaginal disc (C'). Collagen IV is distinguished by the localization of the Vkg::GFP fusion protein. Arrows indicate the basement membranes. Scale bars: 20 μm. Number of biological repeats (N)=3 for all figures. Genotypes: A-B) w^{1118}/Y; UAS-secr.EGFP/+; tub-GAL4/+, GMAP $^{F322A/V325A}$/Y; UAS-secr.EGFP/+; tub-GAL4/+, Atg8a $^{K48A/Y49A}$/Y; UAS-secr.EGFP/+; tub-GAL4/+. C) w^{1118}/Y;P{PTT-un}vkg^{G00454}/+,GMAP $^{F322A/V325A}$/Y;P{PTT-un}vkg^{G00454}/+,
Atg8a $^{K48A/Y49A}$/Y; P{PTT-un}vkg^{G00454}/+.

Supplementary Table S2. The expression level of 29 proteins in mutant Drosophila was higher than that in wild type Drosophila. The proteins are listed by accession number in uniprot, number of protein and peptides, and LFQ (label-free quantification) intensity. These 29 proteins were screened using the following contitions: ratio > 2 and p value < 0.5.

Fasta headers	Number of proteins	Peptides	Razor + unique peptides	Unique peptides	LFQ intensity Atg8a 1	LFQ intensity Atg8a 2	LFQ intensity Atg8a 3	LFQ intensity Atg8a 4	LFQ intensity ATG8a$^{K48A/Y49A}$ 1	LFQ intensity ATG8a$^{K48A/Y49A}$ 2	LFQ intensity ATG8a$^{K48A/Y49A}$ 3	LFQ intensity ATG8a$^{K48A/Y49A}$ 4	LFQ intensity WT1	LFQ intensity WT2	LFQ intensity WT3	LFQ intensity WT4	Average LFQ intensity of Atg8a	Average LFQ intensity of ATG8a$^{K48A/Y49A}$	Average LFQ intensity of WT	Ratio Atg8a/WT	Ratio ATG8a$^{K48A/Y49A}$/WT	p value Atg8a/WT	p value ATG8a$^{K48A/Y49A}$/WT	preferred name	LIR motif	P Anchor
tr|Q7K3E2|Q7K3E2_DROME CG5080, isoform A OS=Drosophila melanogaster GN=CT16297 PE=1 SV=1	1	22	22	22	4.2E+07	9.4E+07	7E+07	5.9E+07	67636000	58255000	29034000	44595000	1.7E+07	2.2E+07	3.6E+07	2.4E+07	66049000	49880000	24680750	2.6761342	2.021008276	0.01159296	0.03557181	CG5080	YES	NO
tr|A0A0B4K6W2|A0A0B4K6W2_DROME Fat facets, isoform E OS=Drosophila melanogaster GN=faf PE=3 SV=	3	18	18	18	8577700	9016300	1.1E+07	1.1E+07	0	8668200	7350900	11604000	0	0	0	0	10005750	6905775	0	#DIV/0!	#DIV/0!	7.5782E-06	0.031221334	faf	YES	NO
tr|Q6IHY5|Q6IHY5_DROME HDC00331 OS=Drosophila melanogaster GN=Dmel\CG34172 PE=1 SV=1	1	2	2	2	1.1E+08	7E+07	7.1E+07	6.4E+07	103160000	87701000	69298000	0	0	0	0	0	78076250	65039750	0	#DIV/0!	#DIV/0!	0.00020042	0.028880452	CG34172	NO	NO
tr|Q8IR72|Q8IR72_DROME FI19011p1 OS=Drosophila melanogaster GN=CG32638-RB PE=1 SV=2	1	3	3	3	1.2E+08	0	9E+07	7.8E+07	61743000	68465000	60786000	0	0	0	0	0	71748250	47748500	0	#DIV/0!	#DIV/0!	0.03018376	0.024545484	CG32638	NO	NO
tr|Q9VDU7|Q9VDU7_DROME LD05707p OS=Drosophila melanogaster GN=Naam PE=2 SV=3	1	7	7	7	1.4E+07	1.5E+07	1.5E+07	1.2E+07	11364000	13802000	13021000	12240000	1.3E+07	0	0	0	13968250	12606750	3127500	4.466267	4.030935252	0.01460035	0.024337065	Naam	NO	NO
tr|Q9VLV9|Q9VLV9_DROME Proctolin OS=Drosophila melanogaster GN=Proc PE=2 SV=2	1	2	2	2	8703600	1.2E+07	8385500	8736000	6878200	4526400	7787400	4594800	3536500	3237600	3024800	0	9499525	5946700	2449725	3.8777924	2.427496964	0.00115312	0.023807031	Proc	NO	NO
tr|A0A0B4KEK7|A0A0B4KEK7_DROME PI31, isoform D OS=Drosophila melanogaster GN=PI31 PE=4 SV=1;s	2	4	4	4	1.1E+07	1.8E+07	1.8E+07	1.2E+07	14105000	17153000	16649000	14322000	1E+07	0	1E+07	8975700	15120500	15557250	7441175	2.0320044	2.090698042	0.04979791	0.021273536	PI31	YES	NO
tr|Q9VIX4|Q9VIX4_DROME Acyl-coenzyme A oxidase OS=Drosophila melanogaster GN=Dmel\CG17544 PE=1	1	20	20	20	4.9E+07	4.3E+07	7.8E+07	8.9E+07	24183000	27366000	17354000	49323000	5441200	8901300	5936100	8904100	64562500	29556500	7295675	8.8494211	4.051235835	0.00210498	0.018795974	CG17544	YES	NO
tr|Q9VZF1|Q9VZF1_DROME CG1309 OS=Drosophila melanogaster GN=Dmel\CG1309 PE=1 SV=1	1	9	9	9	1.6E+07	1.8E+07	1.6E+07	2.4E+07	15907000	23462000	16287000	13676000	9482300	1E+07	0	0	18514250	17333000	4914325	3.7674045	3.527035758	0.0077624	0.012796017	CG1309	NO	NO
sp|P02515|HSP22_DROME Heat shock protein 22 OS=Drosophila melanogaster GN=Hsp22 PE=1 SV=4	1	9	9	9	1.1E+08	1.4E+08	5.8E+07	9.8E+07	17198000	14414000	19554000	9968500	0	7825500	0	6437400	102654000	15283625	3565725	28.789096	4.286260157	0.00137242	0.00707916	Hsp22	YES	NO
tr|A0A0B4LH23|A0A0B4LH23_DROME RIC-3, isoform V OS=Drosophila melanogaster GN=RIC-3 PE=4 SV=1	15	8	8	8	1.8E+07	1.7E+07	1.8E+07	1.4E+07	24466000	16090000	22908000	21190000	0	1.5E+07	0	0	16832750	21163500	3699000	4.5506218	5.721411192	0.01354687	0.005457873	RIC-3	NO	NO
tr|Q9VGE7|Q9VGE7_DROME Beta-galactosidase OS=Drosophila melanogaster GN=Ect3 PE=1 SV=1	1	9	9	9	2.2E+07	1.6E+07	1.7E+07	1.5E+07	25924000	27301000	20987000	16756000	1.2E+07	7952400	9202500	0	17569000	22742000	7319475	2.4003088	3.107053443	0.01395916	0.004790721	Ect3	NO	NO
tr|Q7K3B7|Q7K3B7_DROME LD40177p OS=Drosophila melanogaster GN=CG11208 PE=1 SV=1	1	14	14	14	3.8E+07	1.8E+07	3.5E+07	3.7E+07	21592000	20363000	24913000	28255000	1.2E+07	9489700	0	0	31894750	23780750	5412925	5.8923318	4.393327083	0.00300544	0.002326007	CG11208	YES	NO
tr|Q7K5M6|Q7K5M6_DROME GH04176p OS=Drosophila melanogaster GN=Sip1 PE=1 SV=1	1	9	9	9	1.7E+07	1.2E+07	1.4E+07	1.2E+07	10023000	12230000	15515000	12410000	0	0	0	6741300	13654500	12544500	1685325	8.1019981	7.443371457	0.00147858	0.001739584	Sip1	NO	YES
sp|Q9VG97|GSTD3_DROME Inactive glutathione S-transferase D3 OS=Drosophila melanogaster GN=GstD3 PE=:	1	7	7	7	1.8E+07	1.8E+07	3.1E+07	2.7E+07	14837000	19676000	27681000	20322000	0	6126200	0	0	23691500	20629000	1531550	15.468969	13.4693611	0.00094562	0.000784903	GstD3	YES	NO
tr|Q53XG2|Q53XG2_DROME Division abnormally delayed, isoform B OS=Drosophila melanogaster GN=dally PE	2	7	7	7	6447800	5766300	7550500	5899000	6542700	8155700	5551100	3985100	0	0	0	0	6415900	6058650	0	#DIV/0!	#DIV/0!	4.0691E-06	0.000449452	dally	YES	YES
tr|Q9VBU6|Q9VBU6_DROME RE24638p OS=Drosophila melanogaster GN=CG11857 PE=1 SV=1	1	6	6	1	6.2E+07	7.6E+07	7.1E+07	6.2E+07	53529000	56704000	65897000	51294000	3.1E+07	3.2E+07	2.4E+07	2.5E+07	67521500	56856000	28036750	2.4083212	2.027909797	6.9108E-05	0.000258626	CG11857	YES	NO
sp|P14199|REF2P_DROME Protein ref(2)P OS=Drosophila melanogaster GN=ref(2)P PE=1 SV=2	1	17	17	17	4.6E+08	5.6E+08	3.9E+08	3.5E+08	87102000	68651000	87945000	51836000	7243400	3432200	4237200	2699400	439115000	73883500	4403050	99.729733	16.7800729	9.4014E-05	0.000198945	ref(2)P	YES	YES
sp|Q9VIU7|DPM1_DROME Probable dolichol-phosphate mannosyltransferase OS=Drosophila melanogaster GN=C	1	5	5	5	1.6E+07	1.5E+07	1.6E+07	1.7E+07	16092000	13953000	17980000	9821500	0	0	0	0	16126750	14461625	0	#DIV/0!	#DIV/0!	1.2987E-08	0.000170861	CG10166	YES	NO
tr|Q9W3Q1|Q9W3Q1_DROME GM14286p OS=Drosophila melanogaster GN=Pdp PE=1 SV=1	1	9	9	9	0	1.3E+07	6948500	9111500	6784100	8983800	8088400	5764200	0	0	0	0	7285250	7405125	0	#DIV/0!	#DIV/0!	0.03759869	4.52877E-05	Pdp	YES	NO
tr|Q9VXU2|Q9VXU2_DROME Golgi microtubule-associated protein, isoform A OS=Drosophila melanogaster GN=	2	21	21	21	2.8E+07	2.4E+07	1.8E+07	1.5E+07	13660000	11006000	15479000	10335000	0	0	0	0	21619000	12620000	0	#DIV/0!	#DIV/0!	0.00030631	4.20151E-05	Gmap	YES	YES
tr|M9PCN6|M9PCN6_DROME Numb, isoform D OS=Drosophila melanogaster GN=numb PE=4 SV=1;sp|P16554	2	6	6	6	8211600	1.2E+07	9367700	7753400	6065800	8246400	8399600	6404500	0	0	0	0	9419675	7279075	0	#DIV/0!	#DIV/0!	9.7701E-05	2.04783E-05	numb	NO	NO
tr|Q9VG92|Q9VG92_DROME Glutathione S transferase D8 OS=Drosophila melanogaster GN=GstD8 PE=2 SV=1	1	6	6	6	8031500	8481800	7199800	6258700	7957600	9058900	11393000	8589400	0	0	0	0	7492950	9249725	0	#DIV/0!	#DIV/0!	4.9191E-06	1.72223E-05	GstD8	YES	NO
tr|Q9VN39|Q9VN39_DROME CG9775, isoform A OS=Drosophila melanogaster GN=CG9775 PE=1 SV=1	2	5	5	5	5332800	7418600	0	4784100	6151200	6067800	7774500	5663000	0	0	0	0	4383875	6414125	0	#DIV/0!	#DIV/0!	0.03130612	9.1257E-06	CG9775	NO	YES
tr|A1ZB69|A1ZB69_DROME Glutathione S transferase E4 OS=Drosophila melanogaster GN=GstE4 PE=3 SV=1	1	9	9	9	1.1E+08	1.2E+08	8E+07	9.4E+07	149890000	152980000	141210000	123420000	4.5E+07	4E+07	4.8E+07	4.8E+07	100038250	141875000	45458750	2.2006379	3.120961311	0.00085178	8.33344E-06	GstE4	YES	NO
tr|A4V488|A4V488_DROME Inosine-5-monophosphate dehydrogenase OS=Drosophila melanogaster GN=ras PE=	2	7	7	7	1.2E+07	0	7820000	7853100	11928000	9137300	9035200	9516500	0	0	0	0	6900275	9904250	0	#DIV/0!	#DIV/0!	0.03256616	6.71249E-06	ras	YES	YES
tr|A0A0B4JD21|A0A0B4JD21_DROME Alkylglycerone-phosphate synthase OS=Drosophila melanogaster GN=A1	2	8	8	8	1.9E+07	1.6E+07	1.8E+07	1.8E+07	10879000	12045000	13933000	14633000	0	0	0	0	17562750	12872500	0	#DIV/0!	#DIV/0!	1.5408E-07	5.61398E-06	CG10253	NO	NO
tr|Q86BQ3|Q86BQ3_DROME Uncharacterized protein, isoform A OS=Drosophila melanogaster GN=Dmel\CG132:	3	8	8	7	1.1E+07	8370200	9259500	7679400	9506100	8262800	10817000	8498500	0	0	0	0	9120525	9271100	0	#DIV/0!	#DIV/0!	1.9801E-05	3.86807E-06	CG13284	YES	NO
tr|A0A0B4LGN1|A0A0B4LGN1_DROME Inositol 1,4,5,-tris-phosphate receptor, isoform C OS=Drosophila melan	2	18	18	18	7885300	8429100	8443700	8662200	7844000	7670100	7321500	8500400	0	0	0	0	8355075	7834000	0	#DIV/0!	#DIV/0!	4.04E-09	6.57104E-08	Itp-r83A	YES	NO

Supplementary Table 2 (Related to Figure 2). List of proteins that significantly accumulate in Atg8a ^{KG07569} and Atg8a$^{K48A/Y49A}$ mutants.

Results showing the expression level of 29 proteins that are upregulated in Atg8a ^{KG07569} and Atg8a$^{K48A/Y49A}$ mutants. The proteins are listed by accession number in Uniprot, number of protein and peptides, and LFQ (label-free quantification) intensity. These 29 proteins were screened using the following conditions: ratio > 2 and p value < 0.5.
